# Enhanced net primary productivity across Inner Mongolian grasslands despite diverging stability under climate perturbations

**DOI:** 10.1186/s12870-026-09165-7

**Published:** 2026-06-05

**Authors:** Md Lokman Hossain, Derrick Y. F. Lai

**Affiliations:** Department of Geography and Resource Management, The Chinese University of Hong Kong, Shatin, Hong Kong, New Territories China

**Keywords:** Grassland ecosystems, Biodiversity, Climate extremes, Resistance, Resilience, Ecosystem stability

## Abstract

**Background:**

Understanding ecosystems functioning under frequent climatic perturbations is central to predicting ecosystem stability. While studies suggest that stable ecosystems can buffer against climate extremes, there is no agreement on whether this stability arises from their resistance (ability to absorb shock) to perturbations, resilience (capacity to recover) towards disturbances, or a combination of both. Using annual net primary productivity (NPP) and Standardized Precipitation Evapotranspiration Index (SPEI) and aridity index (AI), we investigated the (i) spatiotemporal changes of NPP, (ii) correlation between NPP, SPEI and AI, and (iii) resistance and resilience of meadow steppe, typical steppe, steppe desert, and desert steppe in Inner Mongolia from 2000–2019.

**Results:**

Despite substantial interannual variability, all grassland types showed an upward trend in NPP, ranging from 1.21 g C m^−2^ yr^−1^ in desert steppe to 4.54 g C m^−2^ yr^−1^ in meadow steppe. The highest NPP (g C m^−2^ yr^−1^) was observed in meadow steppe (251), followed by typical steppe (160), steppe desert (95), and desert steppe (83). NPP increased significantly with increasing SPEI values across all grassland types, and with rising AI in steppe desert and desert steppe. Partial correlation analysis controlling for CO_2_ confirmed that NPP remained significantly and positively correlated with precipitation across all grassland types. Species richness ranged between 9 and 14 in meadow steppe, 7 and 17 in typical steppe and 5–10 in steppe desert. The measured NPP showed an increasing trend with rising species richness across these three grassland types, demonstrating a positive diversity-productivity relationship. Vegetation exhibited lower (higher) resistance against dry (wet) climatic conditions and higher (lower) resilience towards dry (wet) climatic conditions for all grasslands except for desert steppe. Typical steppe, meadow steppe and steppe desert are susceptible to extreme dry climate as these grasslands showed lower resistance against extreme dry climate. However, the higher resilience of these grasslands after extreme dry climate highlights that they recover faster after dry conditions compared to the recovery after wet conditions.

**Conclusions:**

The observed increase in vegetation productivity and differing stability has practical implications for pastoralists, landscape managers, and conservationists for grassland management. The demonstrated positive diversity-productivity relationship suggests maintaining greater species richness could buffer productivity losses during climate extremes, while the NPP-AI correlations in steppe desert and desert steppe offer early warning indicators for drought preparedness.

**Supplementary Information:**

The online version contains supplementary material available at 10.1186/s12870-026-09165-7.

## Background

Understanding how terrestrial ecosystems respond to climatic perturbations (e.g., moderate dry climatic conditions) and climate extremes (e.g., exceptional droughts and periods of heavy precipitation) is a fundamental subject in plant ecology and climate change research [1, 2]. Climate perturbations refer to short- to medium-term deviations (e.g., shortage or surplus of water) from normal climatic conditions that disrupt ecosystem functioning (Table 1, [3]). These events are recurrent but may not exceed ecological tolerance thresholds [4]. In contrast, climate extremes (e.g., extreme dry condition) represent severe, low-frequency events that surpass ecosystem adaptive capacities [1]. While climate perturbations put sub-lethal stress on ecosystem functioning [5], climate extremes—occurring less than once per decade historically (1901–2011) [2]—challenge ecosystem stability [6]. The Intergovernmental Science-Policy Platform on Biodiversity and Ecosystem Services (IPBES) warns that nearly 1 million species are at risk of extinction due to land use changes, resource exploitation, and global climate change [7]. These factors contribute to species loss and ecosystem degradation, highlighting the urgent need for understanding ecosystem dynamics and stability [8, 9]. Fluctuations in the global climate has altered precipitation patterns, accelerating the severity and frequency of extreme wet and dry events [10, 11]. Such climatic perturbations and climate extremes significantly impact ecosystem processes, functioning, and stability [12–14].

**Table 1:** Definition of some mentioned terminology in the study

Variable	Definition	Reference
Ecosystem functioning	Collective (biological, chemical and physical) ecosystem processes that maintain the structure, productivity and sustainability of an ecosystem. For example, aboveground biomass production, and annual net primary productivity	Loreau et al. 2001 [15]
Ecosystem stability	Ecosystem’s capacity to maintain consistent properties and functions despite disturbances in environmental conditions	Isbell et al. 2015 [2], Hossain et al. [4]
Resistance	Ecosystem’s ability to withstand climatic perturbations	Tilman and Downing [16]
Resilience	Ecosystem’s capacity to bounce back towards a climatic disturbance event	
Climate perturbations	Short- to medium term deviations from normal climatic conditions that disrupt ecosystem functioning. For example, SPEI values between > −1.28 to ≤ −0.67 are considered moderate dry climatic conditions	Chu et al. [3]; Isbell et al. [2]
Climate extremes	Severe climatic events that exceed ecosystem tolerance thresholds. For example, extreme dry conditions (SPEI ≤—1.28) are events that occurred less frequently than once per decade over the past 1901–2011	Isbell et al. [2]
Species richness	The number of species within a given area	Moore 2013 [17]
Aridity	The ratio of annual precipitation to potential evapotranspiration. The higher the AI value the higher the moisture conditions in the system	Yan et al. 2025 [18]

Evaluating ecosystem functioning and stability under climate extremes is critically important to: (i) quantify ecosystem vulnerability to changing disturbance regimes, and (ii) develop science-based strategies for sustainable ecosystem management [19, 20]. Ecosystem functioning refers to the integrated biological, chemical, and physical processes that sustain productivity, nutrient cycling, and energy flows within ecosystems (Table 1, [15]). These functions—including net primary production (NPP), decomposition, and carbon sequestration—underpin ecosystem services critical for human well-being [7]. Ecosystem stability, in turn, represents the capacity of ecosystems to maintain their structure and function amid environmental disturbances (Table 1). Ecosystem stability typically assessed using multiple approaches, including resistance (ability to withstand change) resilience (capacity to recover) [2], and temporal consistency in productivity (Xu et al. 2023). Like other terrestrial ecosystems (e.g., forests, and shrublands), grassland ecosystems are vulnerable to increasing frequency of climate extremes and thus it is critical to understand how grasslands respond to climate extremes and recover after disturbances.

Grasslands, covering over 30% of the Earth's terrestrial surface and storing > 10% of global carbon, are among the most climate-sensitive ecosystems [21, 22]. These ecosystems, home to over 11,000 grass species [23], are crucial for maintaining ecological balance and providing vital services, such as recreation and pollination [24]. Shifts in precipitation regime have significant consequences for grassland ecosystem stability [25, 26]. For example, a 9-year experiment in temperate grasslands of northern China demonstrated that aboveground NPP increases with higher precipitation (Xu et al. 2023). Similarly, in Inner Mongolian grasslands, precipitation has been identified as the primary determinant of aboveground biomass production [27]. However, climate change is disrupting this water-dependent balance through more frequent extremes. Where moderate precipitation enhances stability [25, 26], intensified droughts and deluges now threaten productivity across natural and managed grasslands worldwide [4, 28]. This paradox—grasslands being simultaneously sustained by precipitation yet jeopardized by its increasing variability—underscores the urgent need to understand precipitation-productivity thresholds under climate change [29].

Evidence indicates that this vulnerability is becoming increasingly extreme across China [30], where more frequent droughts and deluges threaten to destabilize grassland functioning [31, 32]. Understanding ecosystem stability has therefore become a pressing research priority. While previous research suggests that stable ecosystems exhibit minimal functioning inconsistency and can buffer against climate extremes [2], there remains ongoing debate regarding whether this stability arises from ecosystem’s resilience towards disturbance, its resistance against perturbation, or a combination of both. These two indicators are crucial measures of an ecosystem's health and its responsiveness to climate change [33]. Although these two indicators have been investigated in controlled settings (e.g., experiments and managed grasslands), early efforts yielded inconsistent results [2, 34–37]. For example, a growing body of evidence suggests that ecosystems with higher species richness tend to exhibit greater resistance against and resilience towards extreme events compared to those with lower species richness [34, 38]. Conversely, some empirical studies demonstrate the opposite trend, revealing inconsistencies in the relationship between species richness and ecosystem stability [4, 36].

While high biodiversity is generally associated with increased resistance against environmental changes [2], this relationship does not necessarily extend to resilience [4], particularly when using Net Primary Productivity (NPP) as an indicator of recovery. For instance, a monoculture grassland may demonstrate similar or even greater resilience to droughts compared to a biodiverse ecosystem [19]. For example, a mesocosm experiment [19] reported that monoculture (*Cichorium intybus*) exhibits higher resilience towards drought in terms of root biomass (3.20 kg m^−3^) compared to a four-species community (2.28 kg m^−3^; *C. intybus*, *Lolium perenne*, *Trifolium repens*, and *T. pratense*). This phenomenon may occur when dominant species in monocultures possess broad ecological niches, allowing them to effectively utilize available resources during the stress period [39]. These contradictory findings underscore how methodological differences- in experimental designs (e.g. short-term and long-term), vegetation types (e.g. grasslands or shrublands), and the study types (e.g. managed, natural, and semi-natural grasslands), as well as classifications of climatic conditions [2, 40]- currently limit our ability to generalize stability patterns.

To overcome these limitations, integrating two critical approaches, including standardized climate metrics and long-term data across multiple grassland types, is of critical importance. Remote sensing data provides innovative methods to evaluate ecosystem health across various spatial and temporal dimensions [41–44]. The application of satellite-derived NPP data to assess vegetation functionality and its relationship with climatic variables has expanded across different ecosystems [45, 46]. Despite previous efforts demonstrating the heterogenous interactions of NPP with climate due to the differences in the features of biomes [47–51], a globally consistent climatic conditions classification index can explain the underlying complex interactions of plants with various intensities of extreme events [2, 4, 40, 52–54]. Among commonly used drought indices, such as the Palmer Drought Severity Index (PDSI), Standardized Precipitation Index (SPI), and Standardized Terrestrial Water Storage Index (STI), the Standardized Precipitation Evapotranspiration Index (SPEI) stands out for its ability to detect water deficits and surpluses over varied timescales [2, 4, 52, 55–58]. By incorporating both precipitation and evapotranspiration, SPEI provides a holistic measure of water availability, making it ideal for assessing ecosystem responses to climatic extremes. This adaptability is critical for understanding grassland resistance and resilience, particularly in regions like Inner Mongolia, where climate variability poses escalating threats.

Like grasslands worldwide, which face growing threats from climate change [13], including declining productivity [4], and ecological instability [2], In Inner Mongolian grasslands (meadow steppe, typical steppe, steppe desert and desert steppe) are also under pressure [58, 59]. The steppe desert typically has lower biodiversity, though some species have adapted to thrive in extreme conditions [58–60]. Even more arid than the steppe desert, the desert steppe is characterized by minimal vegetation, primarily consisting of scattered shrubs and a few hardy grasses. This ecotype is highly vulnerable to climatic changes and disturbances due to its limited biodiversity and resource availability. In contrast, meadow steppe, found in areas with higher moisture levels, supports a rich diversity of grasses, rendering it more resistant to disturbances ([58]; Song et al. 2021). Several studies indicated that extreme weather events (e.g., prolonged droughts and intense heatwaves) pose a significant challenge to the grasslands in Inner Mongolia [38, 58, 59]. These events lead to reduced vegetation cover, productivity, and decreased biodiversity, negatively impacting the livelihoods of local herders and the overall ecological balance of the region [61].

Despite documented sensitivities of Inner Mongolian grasslands to climate extremes ([58, 59]; Song et al. 2021), existing research shows inconsistencies, necessitating a more nuanced understanding of how vegetation in Inner Mongolia responds to the increasing frequency and severity of extreme climate events [1, 20]. This study aims to address these gaps by integrating long-term ecological and climate data. Using growing season and annual temperature, precipitation, aridity index (AI), SPEI values alongside annual NPP data, we examined spatiotemporal changes in productivity, relationships between NPP and temperature, precipitation, AI, SPEI, and species richness, as well as the stability of grasslands across Inner Mongolian (typical steppe, meadow steppe desert steppe and steppe desert) over the recent two decades (2000–2019). We chose to focus on these four grassland types because they represent a wide range of precipitation and temperature gradients [58], as well as a significant variation in species and functional diversity ([59]; Song et al. 2021). The following 4 objectives were formulated:

(i) to evaluate the spatiotemporal changes in annual NPP across four grassland types over 2000–2019;
(ii) to assess the relationships between annual NPP and SPEI, AI, temperature, precipitation, and species richness;
(iii) to examine the resistance of grassland ecosystems against moderate and extreme dry and wet climatic conditions; and
(iv) to examine the resilience of grassland ecosystems towards moderate and extreme dry and wet climatic conditions.

The functioning of grassland ecosystems relies on species composition, functional groups, species abundance, species dominance pattern, and abiotic variables (e.g., soil moisture and climatic conditions) [4, 35, 62]. Some species have shallow roots, some have deep roots, while others are adapted to nutrient-poor and resource-rich environments [63]. As species and functional diversity in a particular grassland type is different from other types, their functioning, and stability to climatic perturbations also differ. Thus, the understanding of ecosystem stability to climate extremes based on specific grassland type (e.g., meadow steppe and desert steppe) is crucial for predicting the response of grasslands to changing climate and for sustainably managing grassland biodiversity. The originality of this study lies in its examination of vegetation resistance and resilience across various grassland types instead of treating all Inner Mongolian grasslands as a single entity. This approach provides empirical evidence of ecosystem stability of different grassland types and offers practical insights for land managers regarding sustainable management strategies and policy development.

## Materials and methods

### Description of the study area

**Fig. 1:**
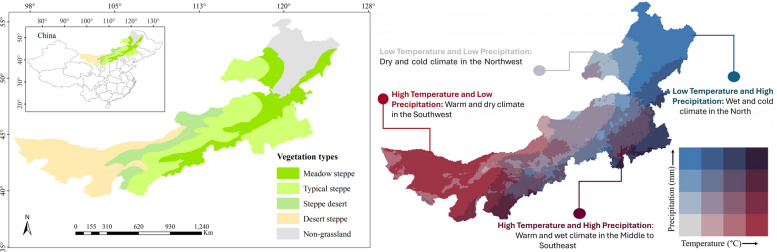
Locations of the studied grassland types (meadow steppe, typical steppe, steppe desert, and desert steppe) and temperature and precipitation gradient in the Inner Mongolia

Inner Mongolia occupies China’s northern frontier, spanning longitudes 97°17'–126°13′E and latitudes 37°42′–53°37′N in a northeast-southwest oriented belt (Fig. 1). Encompassing 1.18 m km2, Inner Mongolia contains the most extensive grassland ecosystems in China. The topography of this region is characterized by high average elevations (> 1,000 m a.s.l.), with a gradual westward and southward elevational gradient [64]. Grasslands in this region feature four distinct types of vegetation [65]. Depending on biophysical conditions (e.g., species composition and climate), these grasslands are classified into typical steppe, desert steppe, steppe desert, and meadow steppe [38]. Characterized by a mix of grasses and herbaceous plants, typical steppe typically experiences moderate rainfall and supports a diverse range of species. It is often dominated by perennial grass. The steppe desert features a more arid climate, with limited rainfall and harsher conditions. Vegetation in this ecotype is sparse and predominantly consists of drought-resistant species, including shrubs and some perennial grasses. Various dominant plant species distinguish the vegetation of each grassland from the others. For instance, desert steppe and steppe desert are distinguished by perennial grasses (e.g., *A. polyrrhizum*), xerophyte herbs (e.g., *Stipa glareosa* and *S. gobica*), and shrubs (e.g., *Caragana sinica and Artemisia xerophytica*), which exhibit remarkable drought resistance due to their deep root systems and water-retaining capacities ([58]; Song et al. 2021). In a recent study in Inner Mongolia (Song et al. 2021), the reported enhanced drought resistance of three dominant species (*S. breviflora, Cleistogenes squarrosa* and *Convolvulus ammannii*) in desert steppe was linked to decreased water consumption, as well as improved water absorption and use efficiency.

*Festucca lenensis*, *S. baicalensis*, *Poa attenuata* and *Leymus chinensis* prevail in meadow steppe, but *Carex duriusula*, *Leymus chinensis*, *S. krylovii* and *S. grandis* predominate in typical steppe [58–60]. The plants found in typical and meadow steppes are mostly herbaceous in composition and low-anchored species, which are low resistant to rising weather variability [59]. The plants found in desert steppe and steppe desert are deep-rooted species that are good at absorbing stresses produced by perturbation [58]. Since species and functional diversity vary among different grassland types, their functioning and stability in response to climatic disturbances also differ, which is evident in other grassland types worldwide [2, 35]. Despite some empirical evidence indicating higher (lower) resistance in desert steppe and steppe desert (typical and meadow steppes) associated with species and functional diversity in Inner Mongolian grasslands ([58, 59]; Song et al. 2021), there is no consensus on whether these species and functional diversity reflect higher or lower resilience after drought or wet events.

**Fig. 2:**
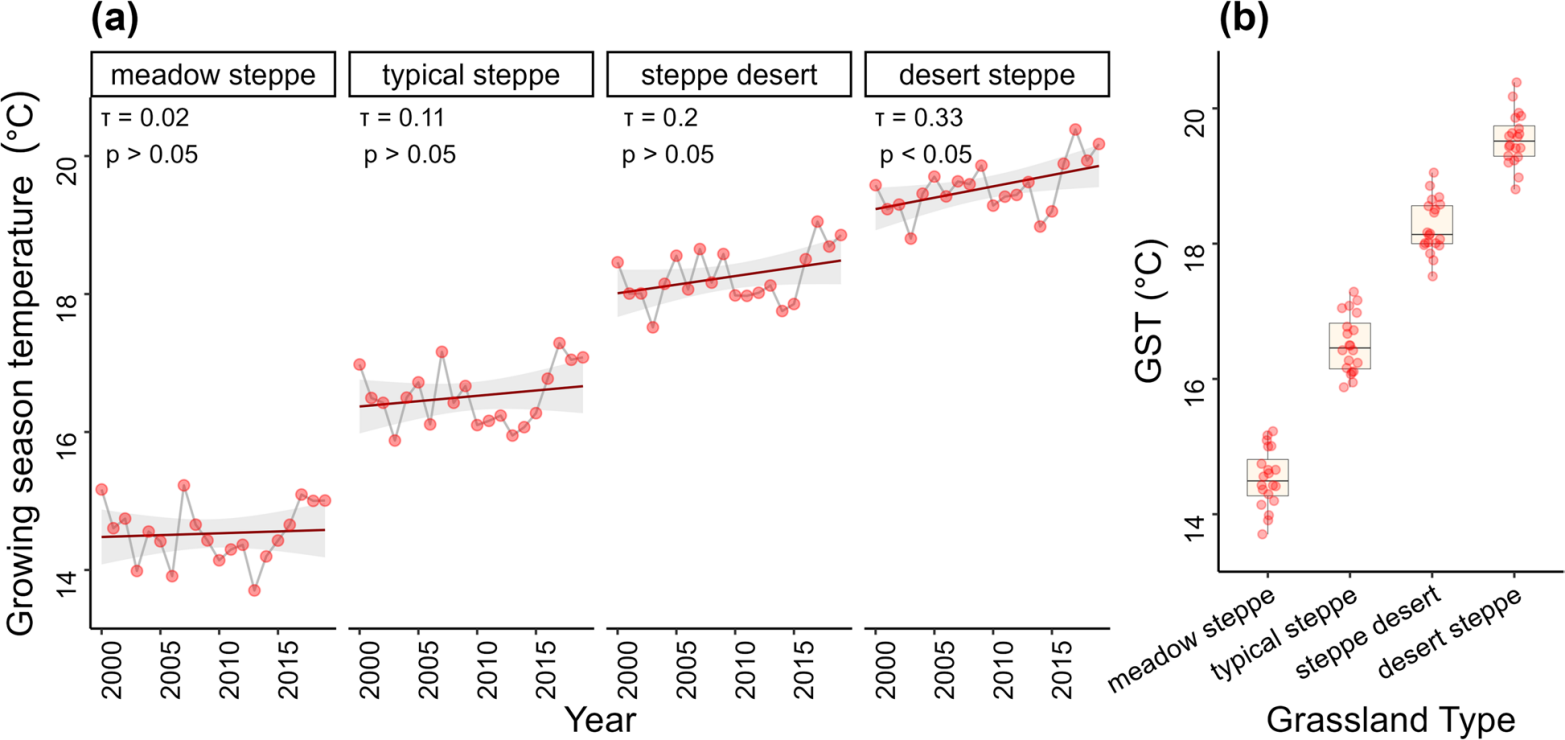
Temporal trends (**a**) and variations (**b**) of growing season temperature (GST) in meadow steppe, typical steppe, steppe desert and desert steppe for the period 2000–2019. Positive tau (τ) values indicate the strength and direction of the monotonic increase in GST over the specified period. The *p* values denote the level of significance of the increasing trends. The solid blue lines represent the trend line of GST. The confidence intervals (95%) for fluctuations in GST are indicated as bands along the lines

**Fig. 3:**
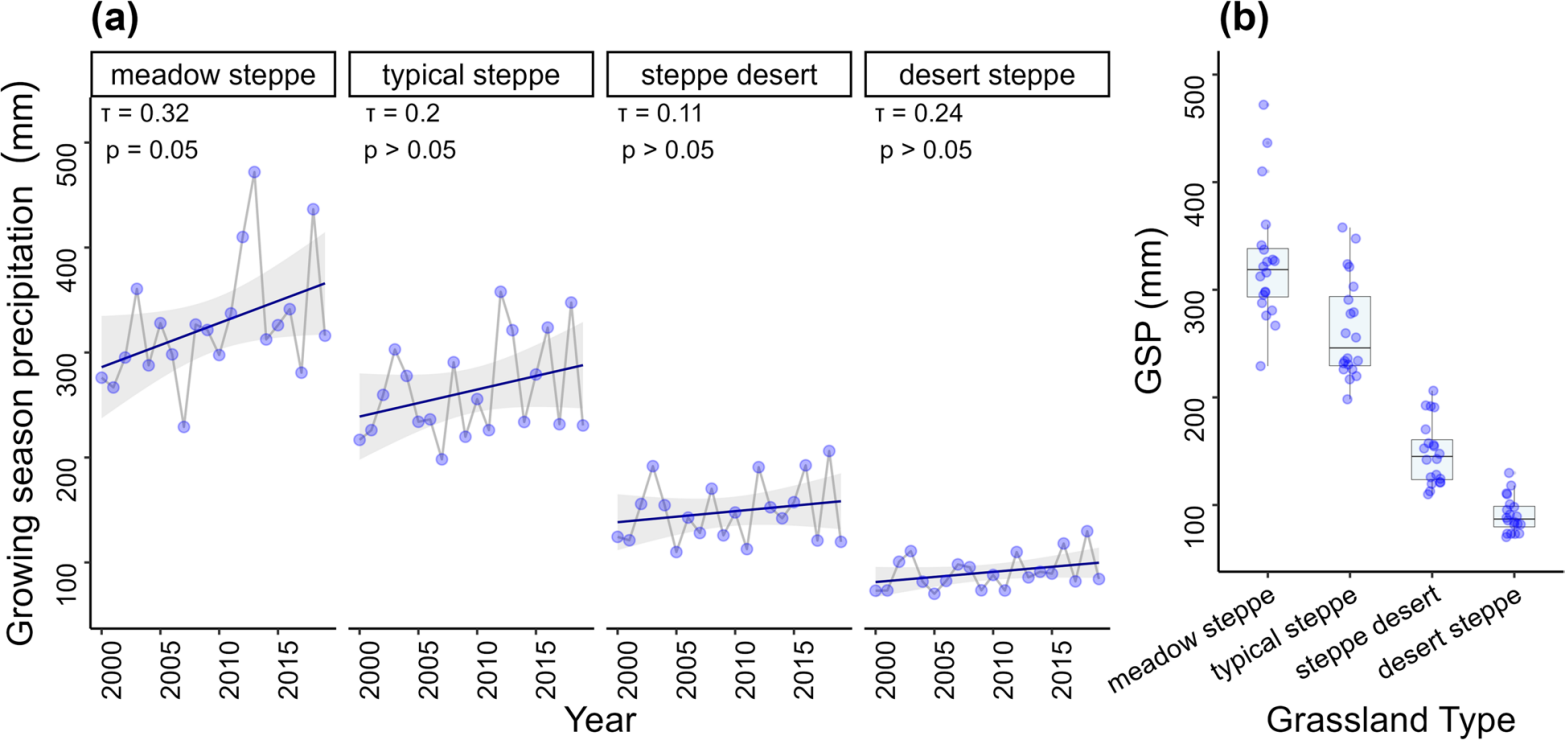
Temporal trends (**a**) and variations (**b**) of growing season precipitation (GSP) in meadow steppe, typical steppe, steppe desert and desert steppe for the period 2000–2019. Positive tau (τ) values indicate the strength and direction of the monotonic increase in GSP over the specified period. The *p* values denote the level of significance of the increasing trends. The solid blue lines represent the trend line of GSP. The confidence intervals (95%) for fluctuations in GSP are indicated as bands along the lines

The annual mean temperature declines from 7.2 °C in desert steppe to 5.1 °C in steppe desert, 3 °C in typical steppe, and 2.2 °C in meadow steppe [58]. The annual precipitation in these grasslands ranges between 45 and 500 mm (increases in the order of desert steppe (45–215 mm) > steppe desert (135–311 mm) > typical steppe (300–400 mm) > meadow steppe (350–500 mm) [58]. Growing season climatic conditions have been reported to influence the functioning of grasslands in Inner Mongolia [64]. The growing season in Inner Mongolia typically extends from April to September [38]. Growing season temperature (GST) and growing season precipitation (GSP) in all grassland types in Inner Mongolia exhibited increasing trends over 2000–2019, of which desert steppe (typical steppe) experienced a significant rise in GST (GSP) (Figs. 2 and 3). Similar rising trends were observed for mean annual temperature (MAT) and annual precipitation (AP) during the same period (Figs. S1 and S2). Mean GSP over 2000–2019 showed a decreasing order from 325.99 mm (meadow steppe) > 263.46 mm (typical steppe) > 148.46 mm (steppe desert) > 99.55 mm (desert steppe) (Fig. 2a). Mean GST across grasslands ranges from 14.53 °C in meadow steppe, 16.52 °C in typical steppe, 18.25 °C in steppe desert and 19.54 °C in desert steppe (Fig. 3b).

### Data sources

**Fig. 4:**
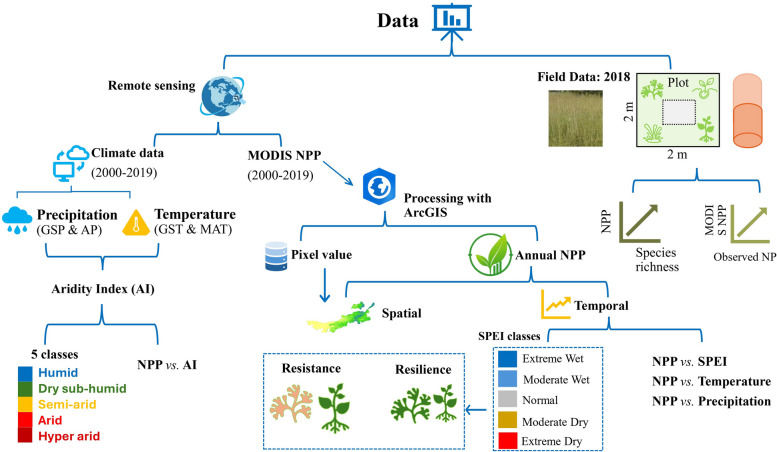
Flowchart of the experimental design and methodology used in the study. NPP: Net primary productivity; GSP: Growing season precipitation; AP: Annual precipitation; GST: Growing season temperature; MAT: Mean annual temperature; AI: Aridity index; SPEI: Standardized precipitation evapotranspiration index

The NPP data for these four grasslands were collected from the Moderate Resolution Imaging Spectroradiometer (MODIS) product (Fig. 4), especially from the gap-filled MOD17A3HGF-Version 6 [66]. Daily land surface temperature (LST) data were sourced from the MODIS (MODIS/061/MOD11A1), which has a spatial resolution of 1 km [67]. Additionally, daily precipitation data were obtained from the Climate Hazards Group InfraRed Precipitation with Stations (CHIRPS; v2.0), featuring a spatial resolution of 5.5 km [68]. The MODIS LST product has been validated as consistent with in situ measurements across different land uses, including grasslands [69]. The CHIRPS precipitation data have been widely used in calculating the SPEI [70]. The SPEI values derived from CHIRPS precipitation data exhibited strong agreement with observation-based SPEI estimates worldwide [71]. Validation of the MODIS NPP with field observation in previous studies [38, 72] has confirmed its accuracy (Fig. 4). Annual CO_2_ concentration data were obtained from the NOAA Global Monitoring Laboratory (GML) Global Greenhouse Gas Reference Network. Specifically, we used the atmospheric CO_2_ dry air mole fractions from the NOAA GML cooperative global air sampling network for the period 2000–2019 [73].

### Data processing

**Fig. 5:**
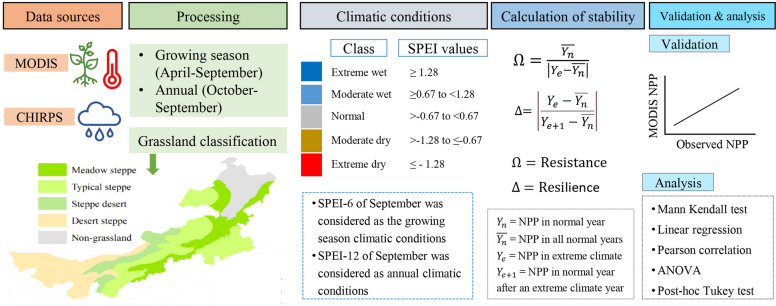
Flowchart illustrating the data sources, processing, classification of climatic conditions, calculation of resistance and resilience, validation and data analysis in the study. MODIS: Moderate Resolution Imaging Spectroradiometer; CHIRPS: Climate Hazards Center InfraRed Precipitation with Stations; SPEI: Standardized Precipitation Evapotranspiration Index; NPP: Net Primary Productivity

Using the MODIS access data tool, raster images of the studied grasslands were collected. ArcGIS 10.1 was used to extract the raster's cell value. To facilitate manipulation, the raster data were converted to TIFF format after being projected to WGS 1984. The MODIS NPP raster spans the years 2000–2019 and has a resolution of 500 m [66]. By removing undesirable inputs from the 8-day leaf area index (LAI) and the portion of photosynthetically active radiation, this data represents a cleaned version of the MOD17 products. The actual levels of NPP were categorized based on grassland types (Fig. 5). The accuracy of the NPP data was validated by assessing the correlation between the MODIS NPP values and the measured NPP at selected sites within three grassland types (steppe desert, and meadow and typical steppes) in 2018 [38]. We conducted field sampling during the peak biomass period in September 2018 (end of growing season) across three grassland types. Our sampling design included 5 representative sites per grassland type, with 3 replicated plots per site (15 plots per grassland type), totaling 45 plots across the study area. At each plot, we first recorded species richness by counting all vascular plant species within a 0.5 × 0.5 m quadrat before harvesting all live aboveground biomass at ground level. For belowground biomass assessment, we extracted soil cores to 20 cm depth and carefully washed the roots to remove soil particles. All biomass samples (both aboveground and belowground) were then oven-dried at 65 °C for 48 h and weighed. We calculated NPP as the sum of these dried aboveground and root biomass measurements for each plot (Fig. 4). Using the LST data obtained from MODIS and precipitation data from CHIRPS, we calculated the SPEI values for 6 months (April-September) and 12 months (October-September) for each grassland type over the period 2000–2019.

### Classification of climatic conditions

SPEI is a meteorological climatic condition index [74] that accounts for both precipitation and potential evapotranspiration. It has been extensively applied to determine the short-term (1 or 2 months) to long-term (2 or 4 years) climatic conditions (e.g., exceptional drought) in a given location [2, 4]. As both annual and growing season (April-September) climate have been proven to have a substantial impact on ecosystem functioning and stability across diverse grasslands [38, 49, 75], we utilized both 6-month (April-September) and 12-month SPEI values in our study. Specifically, we considered SPEI-6 and SPEI-12 values for September each year. SPEI-6 of September indicates the climatic conditions of the preceding 6 months (i.e., April-September), while SPEI-12 of September represents the climatic conditions of the prior 12 months (i.e., October-September). SPEI is standardized, allowing direct comparison of water deficits and surpluses across different locations and time periods. This property makes SPEI ideal for: (i) classifying individual years/seasons into discrete disturbance categories (extreme dry, moderate dry, normal, moderate wet, extreme wet; [2]), and (ii) calculating resistance and resilience metrics, which require identification of discrete disturbance events and post-disturbance recovery periods. The positive SPEI values indicate the moisture surplus (where precipitation exceeds the evaporation and transpiration) in the environment, while negative SPEI values denote a deficit of moisture (where evaporation and transpiration rates exceed precipitation). The SPEI values of 12-month (SPEI-12) and 6-month (SPEI-6) denote dry, normal, or wet conditions during the annual and growing season climate. Following the methodology of Isbell et al [2], SPEI values were grouped into five classes based on their values: extreme dry (≤—1.28), moderate dry (> −1.28 to ≤ −0.67), normal (> −0.67 to < 0.67), moderate wet (≥ 0.67 to < 1.28), and extreme wet (≥ 1.28) (Fig. 5).

**Fig. 6:**
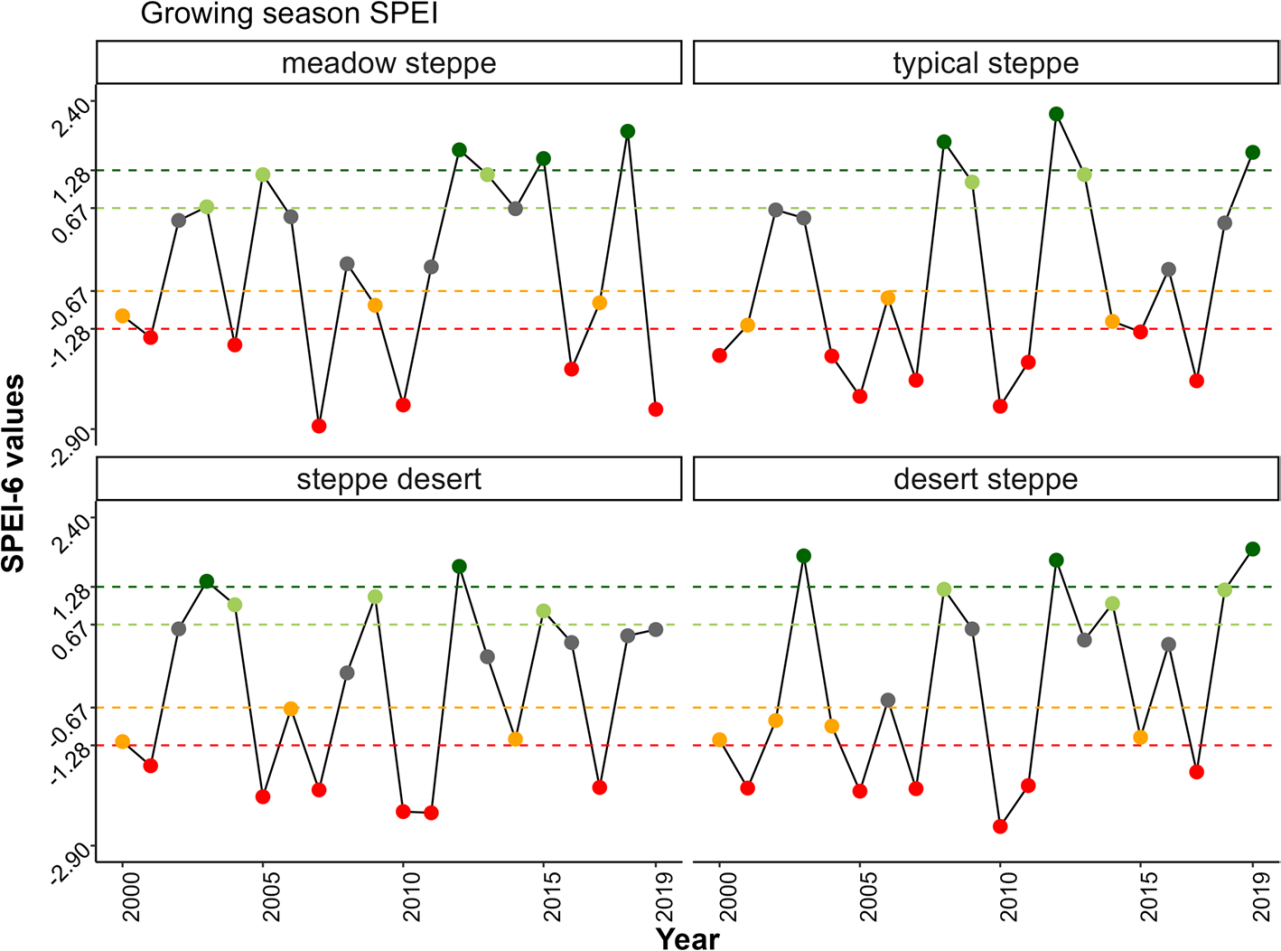
The number of occurrences of growing season climatic events (extreme wet, moderate wet, normal, moderate dry, and extreme dry) based on 6-month (April-September) standardized precipitation evapotranspiration index (SPEI) values for four grassland types (meadow steppe, typical steppe, steppe desert and desert steppe) in Inner Mongolia for the period 2000–2019. The dark olive green and dark green points indicate the moderate wet and extreme wet conditions, while orange and red points denote the moderate dry and extreme dry conditions. Normal climatic conditions are shown in grey points. The horizontal dashed lines indicate the cut-off values of growing season SPEI in respective climatic conditions (dark green for extreme wet, dark olive green for moderate wet, orange for moderate dry, and red for extreme dry). The number of occurrences of annual climatic conditions based on 12-month (October-September) SPEI values have been shown in Fig. S3

For the growing season SPEI values, there were 3 extreme wet, 3 moderate wet, 3 moderate dry, 6 extreme dry and 5 normal climatic events in meadow steppe, 3 extreme wet, 2 moderate wet, 3 moderate dry, 8 extreme dry, and 4 normal climatic events in typical steppe for the period 200–2019 (Fig. 6). In steppe desert (desert steppe), we recorded 2 (3) extreme wet, 3 (3) moderate wet, 3 (4) moderate wet, 6 (6) extreme dry, and 6 (4) normal climatic events during the growing season in the same period (Fig. 6). Likewise, for annual SPEI values, we also recorded similar number of occurrences of climatic conditions for respective grassland types, except for meadow steppe, where 2 moderate dry and 7 extreme dry events were observed (Fig. S3).

### Calculation and classification of aridity

**Fig. 7:**
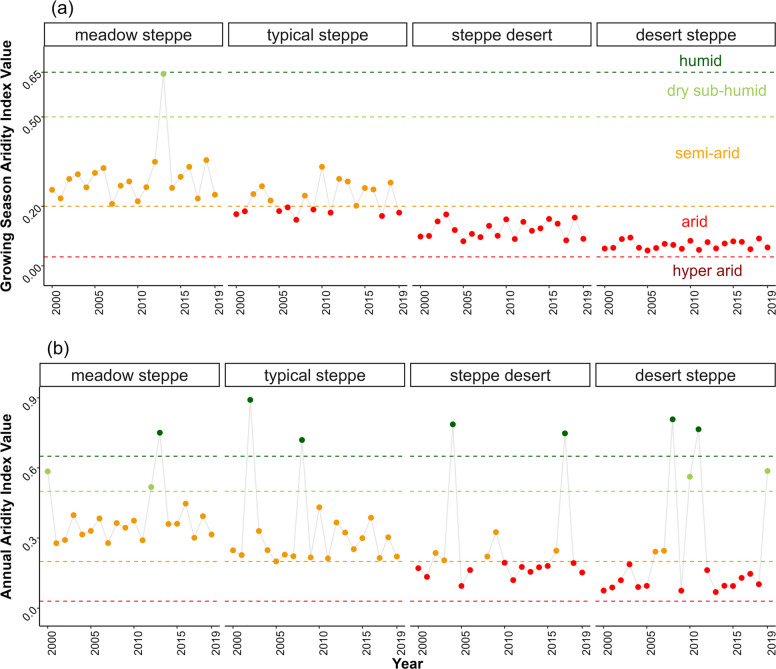
The number of occurrences of growing season (**a**) and annual (**b**) aridity conditions (hyper arid, arid, semi-arid, dry sub-humid and humid) based on 6-month (April-September) and 12-month aridity index (AI) values for four grassland types (meadow steppe, typical steppe, steppe desert and desert steppe) in Inner Mongolia for the period 2000–2019. The dark olive green and dark green points indicate the humid and dry sub-humid conditions, while orange and red points denote the semi-arid and arid conditions. None of the grassland types experienced hyper arid conditions over 2000–2019. The horizontal dashed lines indicate the cut-off values of AI values in respective climatic conditions (dark green for humid, dark olive green for dry sub-humid, orange for semi-arid, and red for arid)

AI is widely used in grassland ecosystems in investigating response of grassland functioning and stability to gradients of aridity [18, 47]. In this study, we used growing season and annual AI to identify climatic conditions in respective grassland types and examine the relationships of annual NPP and growing season and annual aridity. First, employing the Thornthwaite method, we calculated potential evapotranspiration (PET) from monthly mean temperature. Second, we calculated AI values using the ratio of mean precipitation over mean PET (precipitation/PET). For growing season AI values, we considered mean values of April-September, and for annual AI values, we used mean values of January-December. Finally, based on the calculated values, we classified the values into 5 classes, including hyper arid (< 0.03), arid (< 0.03–0.2), semi-arid (< 0.2–0.5), dry sub-humid (0.5–0.65) and humid (> 0.65) [76, 77]. According to this classification [76], all grassland types fall within arid and semi-arid climatic conditions (Fig. 7). Specifically, the growing season AI indicates that steppe desert and desert steppe grasslands belong to arid climatic conditions, while meadow steppes fall within semi-arid conditions (except dry a sub-humid condition in 2013) (Fig. 7a). Typical steppe fluctuated between arid and semi-arid climatic conditions over the study period (Fig. 7a). The annual AI shows similar patterns, with some exceptional wetter years (Fig. 7b). For example, there are three wetter years (2 dry sub-humid and 1 humid condition) in meadow steppe, two wetter years (2 humid conditions) in typical steppe and steppe desert, and 4 wetter years (2 dry sub-humid and 2 humid conditions) in desert steppe (Fig. 7b).

Unlike SPEI, AI is not standardized and retains absolute information about the ratio of precipitation to PET. This property allows us to: (i) classify grassland types along an absolute aridity gradient (arid: AI 0.03–0.2; semi-arid: AI 0.2–0.5; [76]), and (ii) test whether the functional relationship between productivity and water availability differs between ecosystems under chronic vs. intermittent water limitation—a question SPEI cannot address because its standardization removes baseline differences. Specifically, while SPEI mauy whether NPP increases with wetter-than-normal conditions across all grasslands, AI allows us to ask whether NPP continues to increase linearly with absolute water availability in arid grasslands but saturates in semi-arid grasslands, indicating threshold effects in productivity responses. Thus, SPEI and AI serve complementary rather than redundant roles in our analysis.

### Determination of ecosystem stability

Resilience and resistance are two distinct features of ecosystems used to determine various aspects of ecosystem functionality and stability [78]. The capacity of an ecosystem to tolerate the effects of an extreme event is referred to as its resistance, whereas its resilience refers to both its capacity and degree of returning to its pre-disturbed state after a disturbance. A substantial body of research has studied the resistance and resilience of ecosystems using several equations [2, 4, 37, 79, 80]. In this study, we employed the following formulas to calculate resistance (*Equation i*) and resilience (*Equation ii*) of grasslands [2, 4]. Based on these equations, resistance and resilience measures as follows: (1) symmetric, enabling comparison between both positive and negative disturbances; (2) dimensionless, allowing for comparison across various ecosystems; and (3) appropriate for ecosystems that are highly sensitive to stress. It is important to note that the calculations of resistance and resilience presented in this study are applicable only to perennial plant community types.

i $$\mathrm{R}\mathrm{e}\mathrm{s}\mathrm{i}\mathrm{s}\mathrm{t}\mathrm{a}\mathrm{n}\mathrm{c}\mathrm{e} \left(\Omega \right)=\frac{\overline{{Y}_{n}}}{\left|{Y}_{e}-\overline{{Y}_{n}}\right|}$$

ii $$\mathrm{R}\mathrm{e}\mathrm{s}\mathrm{i}\mathrm{l}\mathrm{i}\mathrm{e}\mathrm{n}\mathrm{c}\mathrm{e} \left(\Delta \right)=\left|\frac{{Y}_{e}-\overline{{Y}_{n}}}{{Y}_{e+1}-\overline{{Y}_{n}}}\right|$$

where $\overline{{Y}_{n}}$ is the NPP value during regular (normal) climatic conditions (i.e. SPEI values range between −0.67 and < 0.67), ${Y}_{e}$ is the NPP value during a disturbance event, and Y_e+1_ is the NPP value during a regular year after a disturbance event. For two (three) consecutive extreme growing seasons or years, the normal growing season or year was considered Y_e+2_ (Y_e+3_).

For example, for calculating the $$\overline{{Y}_{n}}$$ of the growing season in meadow steppe, the mean NPP values during 5 normal climatic conditions (2002, 2006, 2008, 2011, and 2014) were considered (Fig. 6). This approach was followed for other grassland types both for growing season (Fig. 6) and annual (Fig. S3) climatic conditions. We then considered NPP values for the respective moderate and extreme dry and wet conditions, which is defined as disturbance event ($${Y}_{e})$$. For example, we considered NPP values in the years 2003, 2005, and 2013 (2012, 2015 and 2018) as growing season moderate (extreme) wet climatic conditions for meadow steppe. Similar approaches were applied for other grassland types for growing season and annual climatic conditions.

We utilized formula Y_e+1_ to calculate the NPP under normal climatic condition after a subsequent year (growing season) dry/wet event. For instance, we considered the NPP in 2008 following the growing season extreme dry event in 2007 for the meadow steppe. In cases of two consecutive dry or wet events, we employed the formula Y_e+2_, meaning that the NPP in a normal year was considered following two consecutive dry or wet years. This approach was consistently applied across all grassland types for both growing season and annual climatic conditions.

Our study focused on ecosystem responses to annual and growing season climate extremes, which are ecologically relevant timescales for grassland productivity. The resistance and resilience metrics we employed [2] were specifically developed for annual-scale data, as they require: (i) identification of discrete disturbance events based on standardized climate indices, and (ii) comparison of annual NPP during normal vs. extreme years. Finer temporal resolution would capture short-term physiological responses (e.g., stomatal closure, photosynthetic downregulation) rather than the ecosystem-level stability properties that were the focus of our investigation. Furthermore, SPEI values at monthly scales exhibit high temporal autocorrelation, making independent event identification problematic and potentially inflating Type I error rates.

### Data analysis

We evaluated the distributions of annual NPP values through both graphical and statistical assessments. Visual inspection of the histogram revealed a symmetric, bell-shaped distribution, while the quantile–quantile (Q-Q) plot demonstrated close alignment of the data points with the theoretical normal distribution line (Fig. S4). These visual assessments were corroborated by a Shapiro–Wilk normality test [81], confirming that the data did not significantly deviate from a normal distribution (W = 0.99, *p* > 0.05). The temporal trend of NPP in each grassland type as well as across all grasslands combined, were detected using ‘*linear regression*’ in the ggplot2 package in the statistical software R v4.2.0 [82]. The linear regression line was fitted to the data points using the function ‘*geom_smooth (method* = *lm)*’. The significance (*p* values) of the attained upward or downward trends was determined using the Mann–Kendall (MK) test [83]. The tau (*τ*) values obtained from the MK test indicate the strength and direction of the changes in NPP values over 2000–2019. In this case, the NPP values served as the dependent variable, while the year was treated as the independent variable. Linear regression was also employed to assess the relationship between measured NPP and species richness in three grassland types (meadow steppe, typical steppe and steppe desert) for the year 2018.

Before assessing the correlation between NPP and SPEI, we evaluated the autocorrelation of the residuals using the Durbin-Watson test to ensure that the model's residuals met the assumption of independence (Table S1), indicating no autocorrelation [84]. Pearson’s correlation analysis [85] was employed to assess the relationship between annual NPP and both growing season and annual SPEI and AI values across the four grassland types. The resulting *correlation coefficients (R)* and *p* values, obtained from the ‘*stat_cor()*’ function, indicate the strength and direction of the relationship, as well as its significance level. In this analysis, SPEI and AI values were treated as independent variable, while NPP was the dependent variable. A linear regression line was fitted to the data points of the correlation using the ‘*geom_smooth (method* = *lm)*’ function.

Likewise, we conducted a Pearson correlation analysis between NPP and SPEI in normal seasons/years (i.e. SPEI values between > −0.67 and < 0.67) to assess whether NPP was influenced by temporal changes or preceding dry or wet events. Our analysis revealed that there were no significant relationships between annual NPP and SPEI values in these normal seasons/years (Fig. S5). This suggests that NPP values remain stable during these normal periods, allowing us to proceed with our evaluations of resilience without necessitating additional statistical corrections.

Pearson’s correlation analysis was employed to assess the correlation between the annual NPP and growing season and annual climatic variables (GST_max_, GST_mean_, GST_min_, GSP, MAT_max_, MAT_mean_, MAT_min_, and AP [86]. A correlation matrix was used to visualize the correlation. The significance level (*p* < 0.05) of the correlation was shown in asterisk (*), while insignificant correlations (*p* > 0.05) were left black.

To evaluate whether observed NPP-precipitation relationships were confounded by rising atmospheric CO_2_ concentration (369 to 411 ppm over 2000–2019), we conducted partial correlation analyses [87] controlling for CO_2_ effects. For each grassland type, simple Pearson correlations were first calculated between NPP and two precipitation metrics: GSP and AP. Subsequently, partial correlations were computed between NPP and each precipitation metric while controlling for annual mean CO_2_ concentration. Partial correlation coefficients were derived from the residuals of linear regressions with the CO_2_ effects being removed from both NPP and precipitation variables.

One-way Analysis of Variance (ANOVA) was utilized to identify the significant variances in the mean resistance of a grassland type among four intensities (i.e. moderate and extreme dry and wet) of annual and growing season climate extremes [88]. A *post-hoc* Tukey's Honest Significant Difference (HSD) test was carried out to detect the pair-wise analysis of vegetation resistance between different climate extreme intensities [89]. This *post-hoc* test was undertaken because the changes in grassland resistance across the four intensities were found to be statistically significant. A similar method was applied for the analysis of the resilience of NPP towards annual and growing season climatic conditions (i.e., extreme wet, moderate wet, moderate dry, and extreme dry). The post hoc test was not conducted for pairwise comparison when the variance in mean resistance among the four annual climatic extreme events was not significant (*p* > 0.05) in both typical steppe and steppe desert.

To address the limited number of extreme events, we conducted complementary robustness analyses. First, *post-hoc* power analysis using observed effect sizes (Cohen's d) revealed a mean statistical power of 93.6% for resistance comparisons (Table S2) and 90.4% for resilience comparisons (Table S3) across all grassland types [90]. For the key comparison of extreme dry vs. extreme wet conditions, power exceeded 0.80 for all grassland types, indicating adequate sample sizes to detect the large effect sizes present in our data. Moreover, leave-one-out sensitivity analysis [91] was conducted by sequentially removing each event year and recalculating mean resistance and resilience to assess the influence of individual observations on overall conclusions. Removing any single event year changed mean resistance and resilience values by only 5.2%, and 3.6%, respectively, and qualitative conclusions remained unchanged in 97% of analyses for resistance and 93.8% of analyses for resilience (Table S4), indicating that results are not driven by outlier events. All statistical analyses were carried out in the statistical software R v4.2.0 [82].

## Results

### Changes in annual NPP

**Fig. 8:**
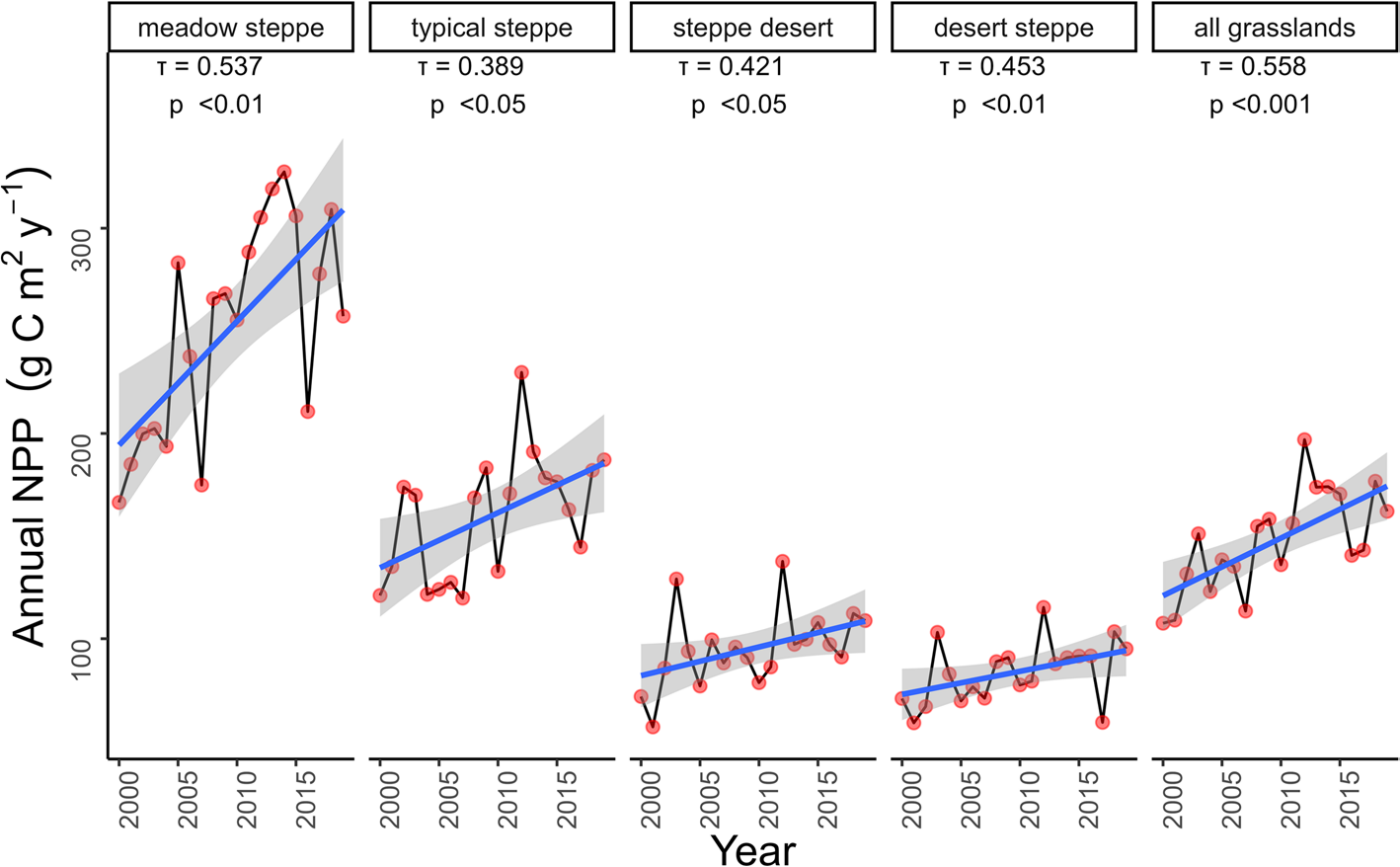
Variations of annual net primary productivity (NPP) in Inner Mongolian grasslands over 2000–2019. Points represent mean NPP values in years in respective grassland types. Each point is the mean NPP of pixel-level values in each year in respective grassland types. Positive tau (τ) values indicate the strength and direction of the monotonic increase in annual NPP over the specified period. The *p* values denote the level of significance of the increasing trends obtained by the Mann–Kendall trend test. The solid blue lines represent the trend line of annual NPP obtained by linear regression. The confidence intervals (95%) for fluctuations in annual NPP are indicated as bands along the lines

**Fig. 9:**
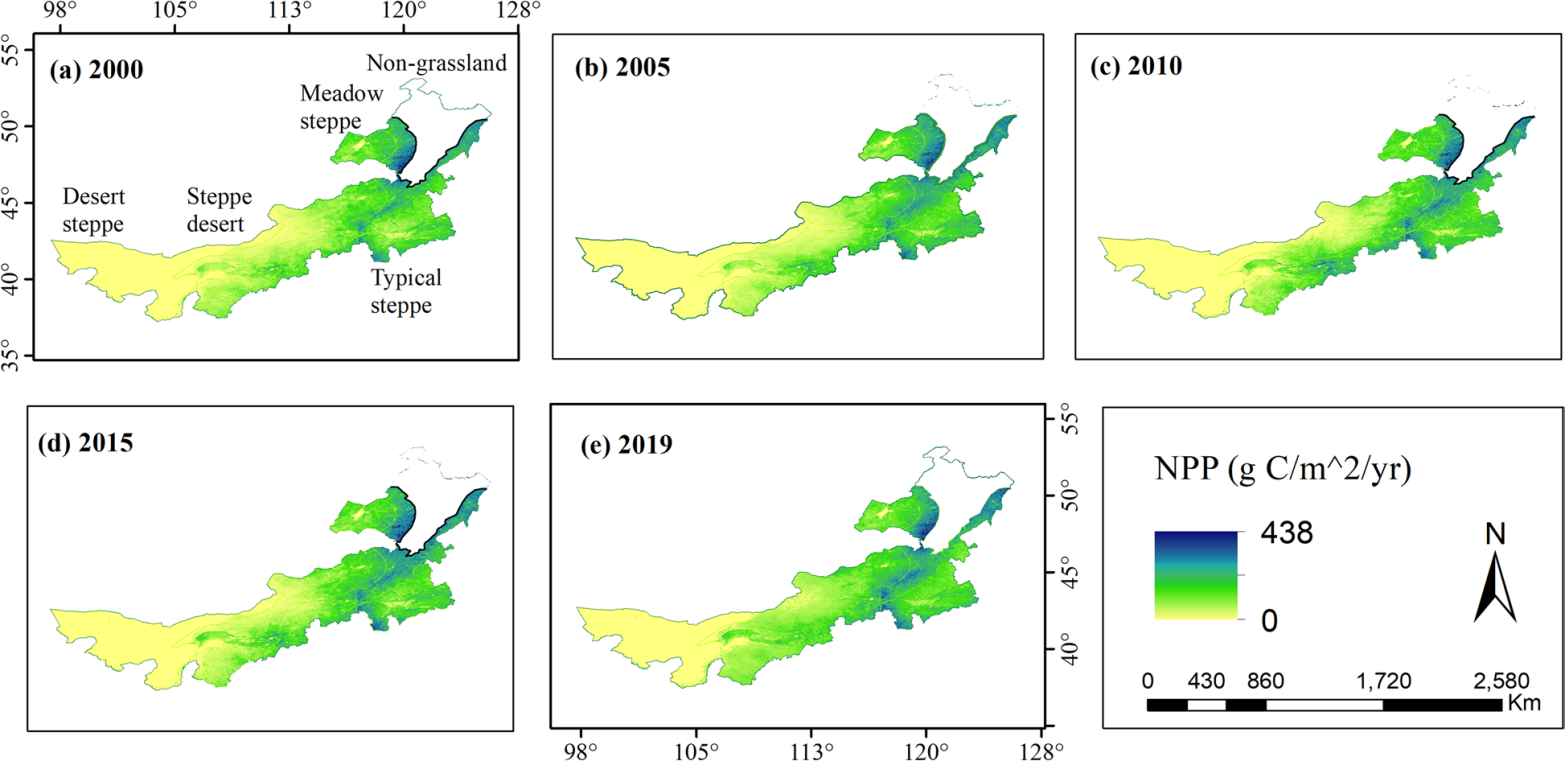
Changes in annual net primary productivity (NPP; g C m^−2^ yr^−1^) across four grassland types (meadow steppe, typical steppe, steppe desert, and desert steppe) in Inner Mongolia over the past 2 decades: **a** 2000, **b** 2005, **c** 2010, **d** 2015, and **e** 2019. Non-grassland areas in Inner Mongolia have been omitted from the spatial analysis

Despite large interannual variations in annual NPP, grasslands in Inner Mongolia showed an overall upward trend in productivity from 2000 to 2019 (Fig. 8). Specifically, significant rising trends (all *p* < 0.05) were observed for the meadow steppe (τ = 0.537), typical steppe (τ = 0.389), steppe desert (τ = 0.421), desert steppe (τ = 0.453) and for all grassland types combined (τ = 0.558). The meadow steppe had the highest annual NPP (251.65 g C m^−2^ yr^−1^) followed by typical steppe (160.03 g C m^−2^ yr^−1^), steppe desert (95.27 g C m^−2^ yr^−1^) and desert steppe (83.55 g C m^−2^ yr^−1^). The mean annual NPP in all grassland types was 147.63 g C m^−2^ yr^−1^, with an increasing rate of 2.726 g C m^−2^ yr^−1^ (Fig. 8). The growth rate of annual NPP (g C m^−2^ yr^−1^) followed the order of meadow steppe (4.54) > typical steppe (3.31) > steppe desert (1.85) > desert steppe (1.21). Spatial changes in NPP across the four grassland types over five periods (2000, 2005, 2010, 2015, and 2019) also showed an upward trend (Fig. 9).

### Relationship between NPP and climatic conditions

#### NPP and climate

**Fig. 10:**
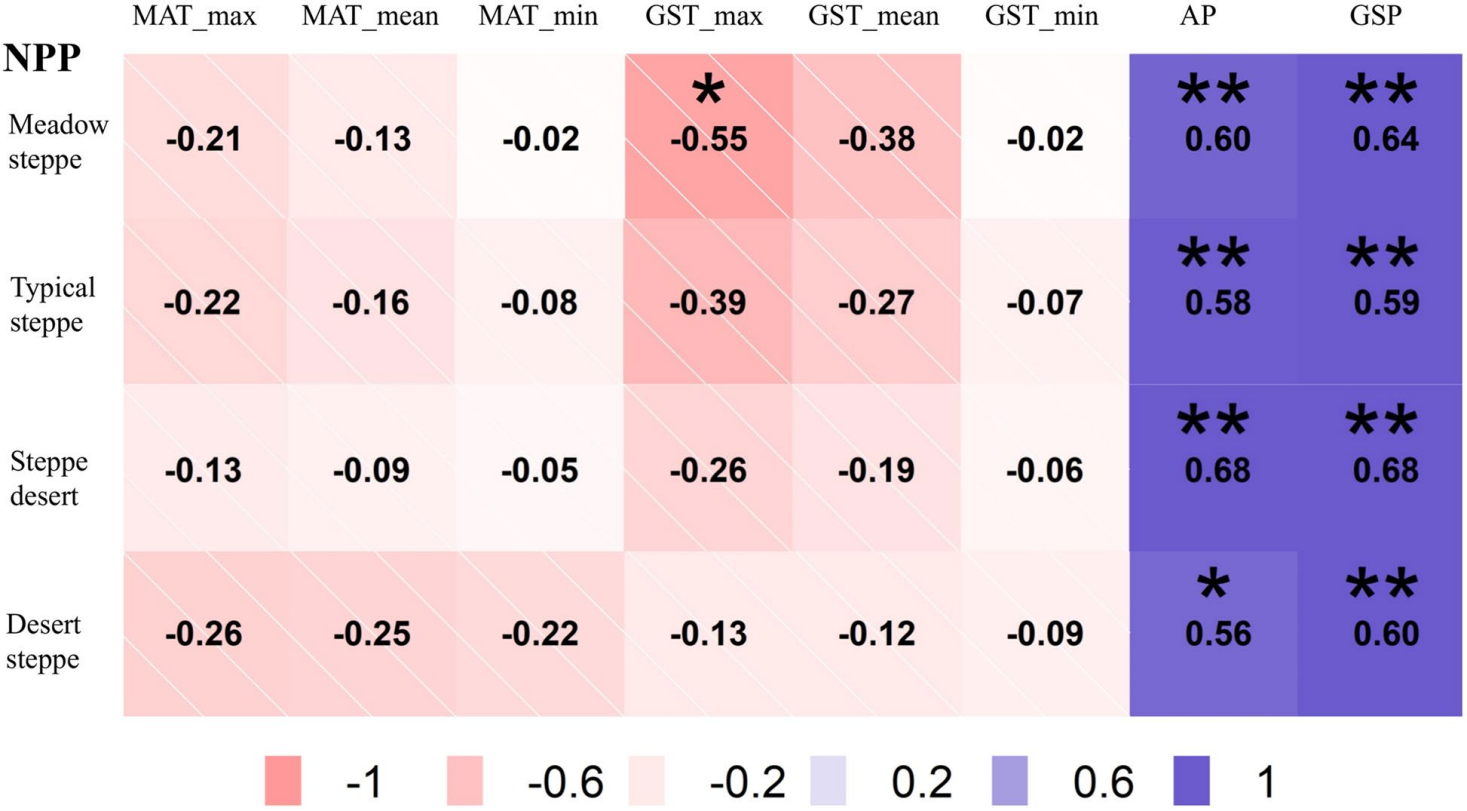
Pearson **c**orrelation between annual NPP and climatic variables. MAT_max_: Mean Annual Maximum Temperature, MAT_mean_: Mean Annual Temperature, MAT_min_: Mean Annual Minimum Temperature, GST_max_: Growing Season Maximum Temperature, GST_mean_: Growing Season Mean Temperature, GST_min_: Growing Season Minimum Temperature, AP: Annual Precipitation, and GSP: Growing Season Precipitation. The significant correlations between NPP and climate variables are marked with asterisk (* for *p* < 0.05, and ** for *p* < 0.01), while insignificant correlations (*p* > 0.05) are left blank. The correlation coefficients are shown with a color gradient ranging from slate blue (*r* = + 1, strong positive correlation) to white (*r* = 0, no correlation) to pastel red (*r* = −1, strong negative correlation)

Across all grassland types, the annual NPP showed negative correlations with all temperature variables, of which meadow steppe exhibited significant negative correlation between NPP and GST_max_ (*r* = −0.55, *p* < 0.05) in meadow steppe (Fig. 10). Conversely, NPP demonstrated significant positive correlations with growing-season and annual precipitation in all grassland types (all *p* < 0.05, Fig. 10).

#### NPP and precipitation after CO_2_ control

**Fig. 11:**
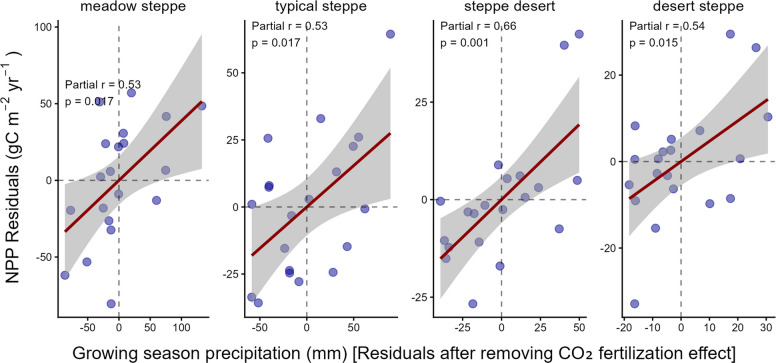
Partial regression plots of the relationship between net primary productivity (NPP) and growing season precipitation (GSP) after controlling for atmospheric CO_2_ concentration across four grassland types in Inner Mongolia (2000–2019). Each panel shows the residuals of NPP after removing the effect of CO_2_ plotted against the residuals of GSP after removing the effect of CO_2_. The solid red line represents the linear regression fit, with shaded bands indicating 95% confidence intervals. Partial correlation coefficients (r) and *p*-values are displayed in each panel

**Fig. 12:**
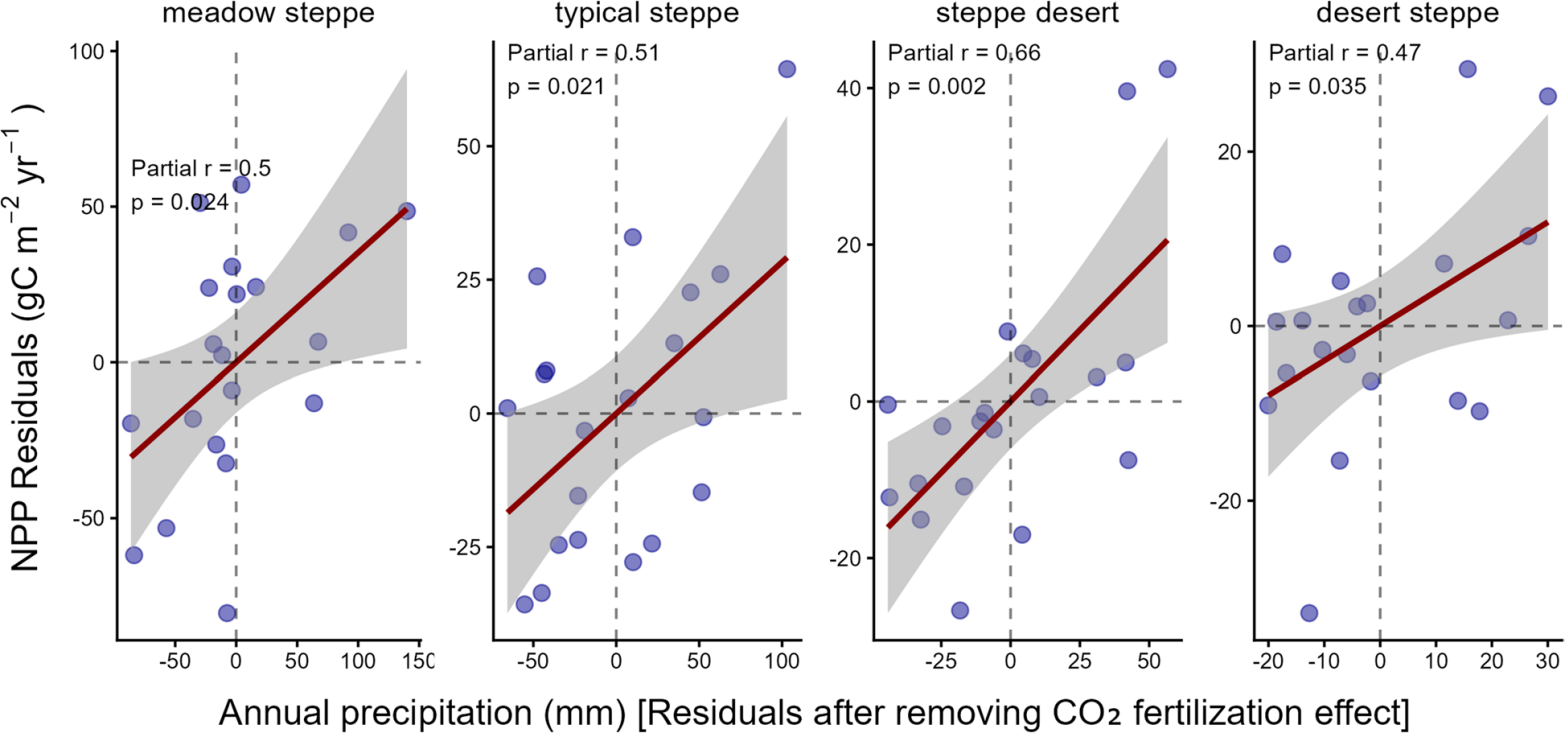
Partial regression plots of the relationship between net primary productivity (NPP) and annual precipitation (AP) after controlling for atmospheric CO_2_ concentration across four grassland types in Inner Mongolia (2000–2019). Each panel shows the residuals of NPP after removing the effect of CO_2_ plotted against the residuals of AP after removing the effect of CO_2_. The solid red line represents the linear regression fit, with shaded bands indicating 95% confidence intervals. Partial correlation coefficients (r) and *p*-values are displayed in each panel

Given the concurrent rise in atmospheric CO_2_ concentration (from 369 ppm in 2000 to 411 ppm in 2019) and NPP across all grassland types (Fig. 8), we conducted partial correlation analyses to determine whether observed NPP-precipitation relationships persisted after accounting for CO_2_ fertilization effects (Figs. 11 and 12). Both GSP and AP were analyzed to compare their relative strengths as predictors of NPP. After controlling for rising atmospheric CO_2_, our results of partial correlation analysis revealed that NPP remained significantly correlated with both GSP (Fig. 11) and AP (Fig. 12) across all grassland types (all *p* < 0.05). Partial correlations for GSP ranged from *r* = 0.53 in meadow steppe to *r* = 0.66 in steppe desert (Fig. 11), while for AP, partial correlations ranged from *r* = 0.47 in meadow steppe to *r* = 0.658 in steppe desert (Fig. 12). GSP consistently outperformed AP as a predictor, with steppe desert showing the strongest water dependency (Fig. 11).

#### NPP and SPEI

**Fig. 13:**
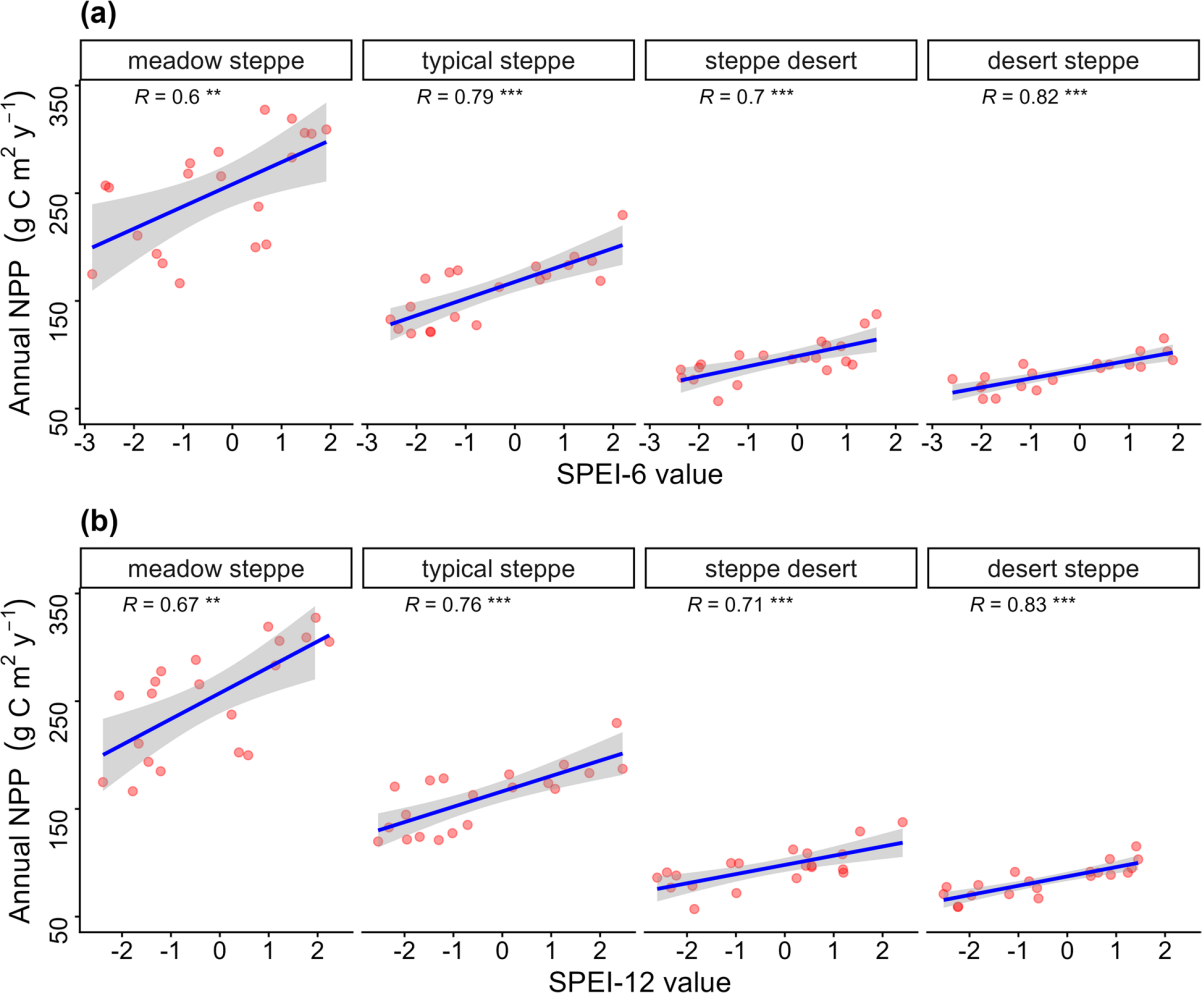
Correlation between annual net primary productivity (NPP) and growing season (**a**) and annual (**b**) standardized precipitation evapotranspiration index (SPEI) for the four grassland types (meadow steppe, typical steppe, steppe desert, and desert steppe) in Inner Mongolia for the period 2000–2019. Correlation coefficients (*R* values) obtained from Pearson correlation analysis denote the strength and direction of the linear relationship between annual NPP and SPEI values. The asterisks (**, and ***) indicate the level of significance (*p* < 0.01, and *p* < 0.001) of the relationships for respective grassland types. The confidence intervals (95%) for relationships are indicated as bands along the solid blue lines

Pearson correlation analysis revealed that the annual NPP significantly increased with wetter growing season conditions (i.e. rising positive SPEI values) for meadow steppe (*R* = 0.60), typical steppe (*R* = 0.79), steppe desert (*R* = 0.70), and desert steppe (*R* = 0.82) (all *p* < 0.01; Fig. 13a). This positive correlation was also observed between the annual NPP and annual SPEI values across all grassland types (all *p* < 0.01, Fig. 13b). This indicates that NPP increases universally during wetter-than-normal years, irrespective of the grassland's baseline aridity.

#### NPP and aridity

**Fig. 14:**
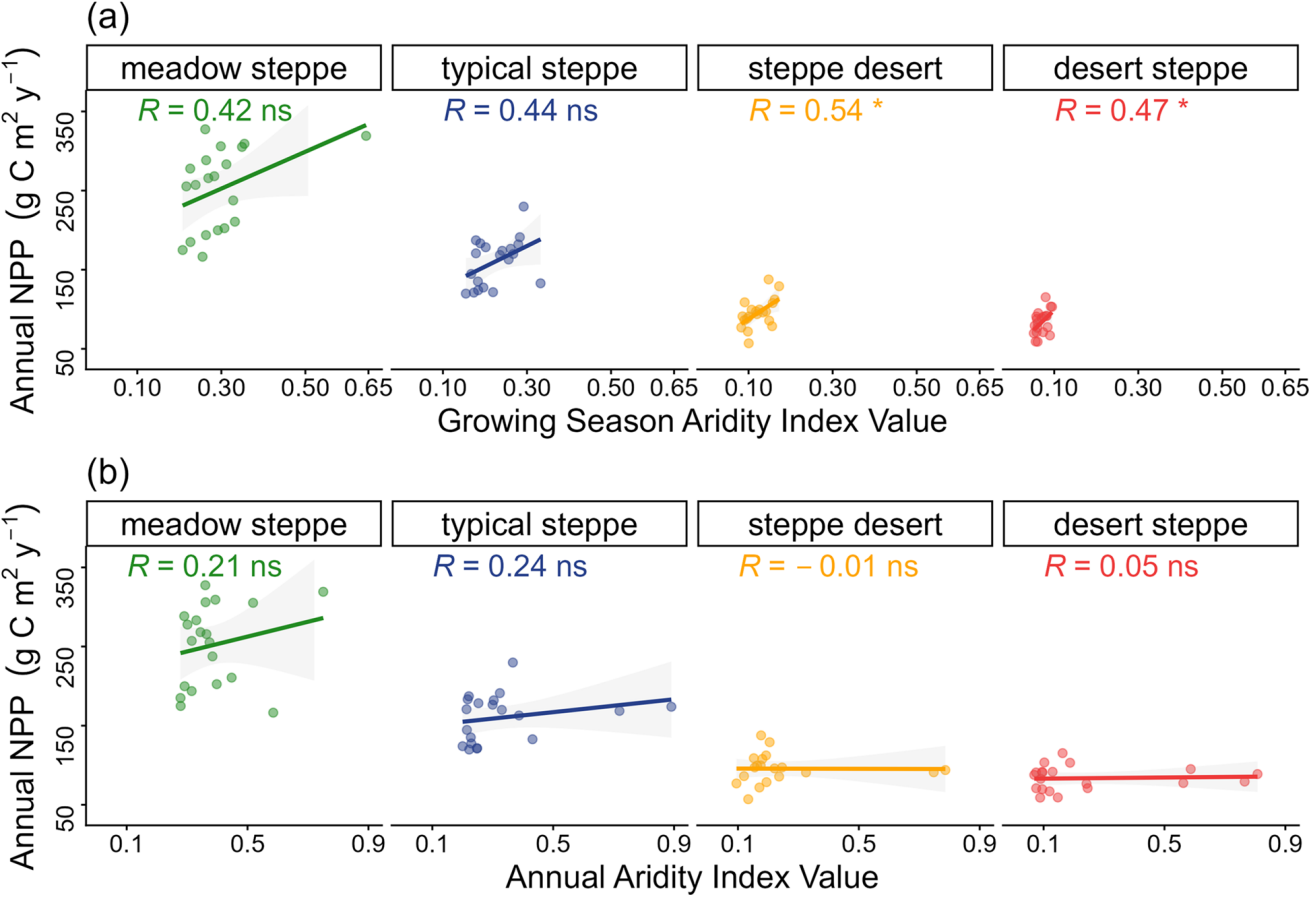
Correlation between annual net primary productivity (NPP) and growing season (**a**) and annual (**b**) aridity index (AI) for the four grassland types (meadow steppe, typical steppe, steppe desert, and desert steppe) in Inner Mongolia for the period 2000–2019. Correlation coefficients (*R* values) obtained from Pearson correlation analysis denote the strength and direction of the linear relationship between annual NPP and AI values. The asterisks (*) indicate the level of significance (*p* < 0.05) of the relationships for respective grassland types. The confidence intervals (95%) for relationships are indicated as bands along the solid blue lines

AI exposed divergent responses between arid and semi-arid grasslands (Fig. 14). Annual NPP in arid grasslands (steppe desert and desert steppe) exhibited significant positive correlations with growing season AI values (steppe desert: *r* = 0.54, *p* < 0.05; desert steppe: *r* = 0.47, *p* < 0.05; Fig. 14a). In contrast, annual NPP in semi-arid grasslands (meadow steppe and typical steppe) did not show any significant correlations with the growing season AI (Fig. 14a) and annual AI (Fig. 14b). This divergence revealed that chronically water-limited ecosystems remained linearly responsive to growing season moisture increases, whereas semi-arid grasslands approached optimal moisture thresholds where additional water yielded diminishing returns.

### Relationship between species richness and NPP

**Fig. 15:**
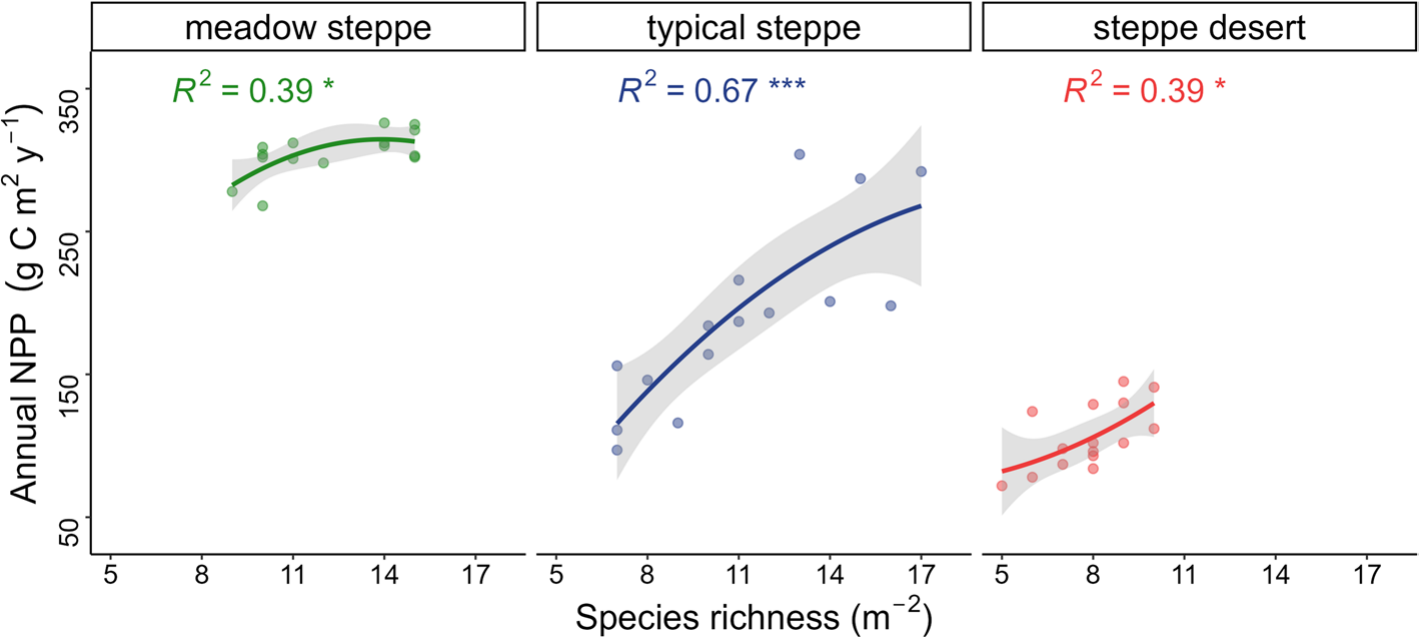
Relationship between species richness and annual net primary productivity (NPP) in meadow steppe, typical steppe and steppe desert for the year 2018. Points are the plot-level values. Lines indicate generalized linear model fits between species richness and annual NPP. Bands represent 95% confidence intervals. Asterisks (* and ***) and R^2^ values indicate the significance (*p* < 0.05, and *p* < 0.001) of the relationship and variation of annual NPP

The measured annual NPP showed significant increasing trend with increasing species richness in three grassland types: meadow steppe (*r*^2^ = 0.39, *p* < 0.05), typical steppe (*r*^2^ = 0.67, *p* < 0.001) and steppe desert (*r*^2^ = 0.39, *p* < 0.05) (Fig. 15).

### Ecosystem resistance against climate extremes

**Fig. 16:**
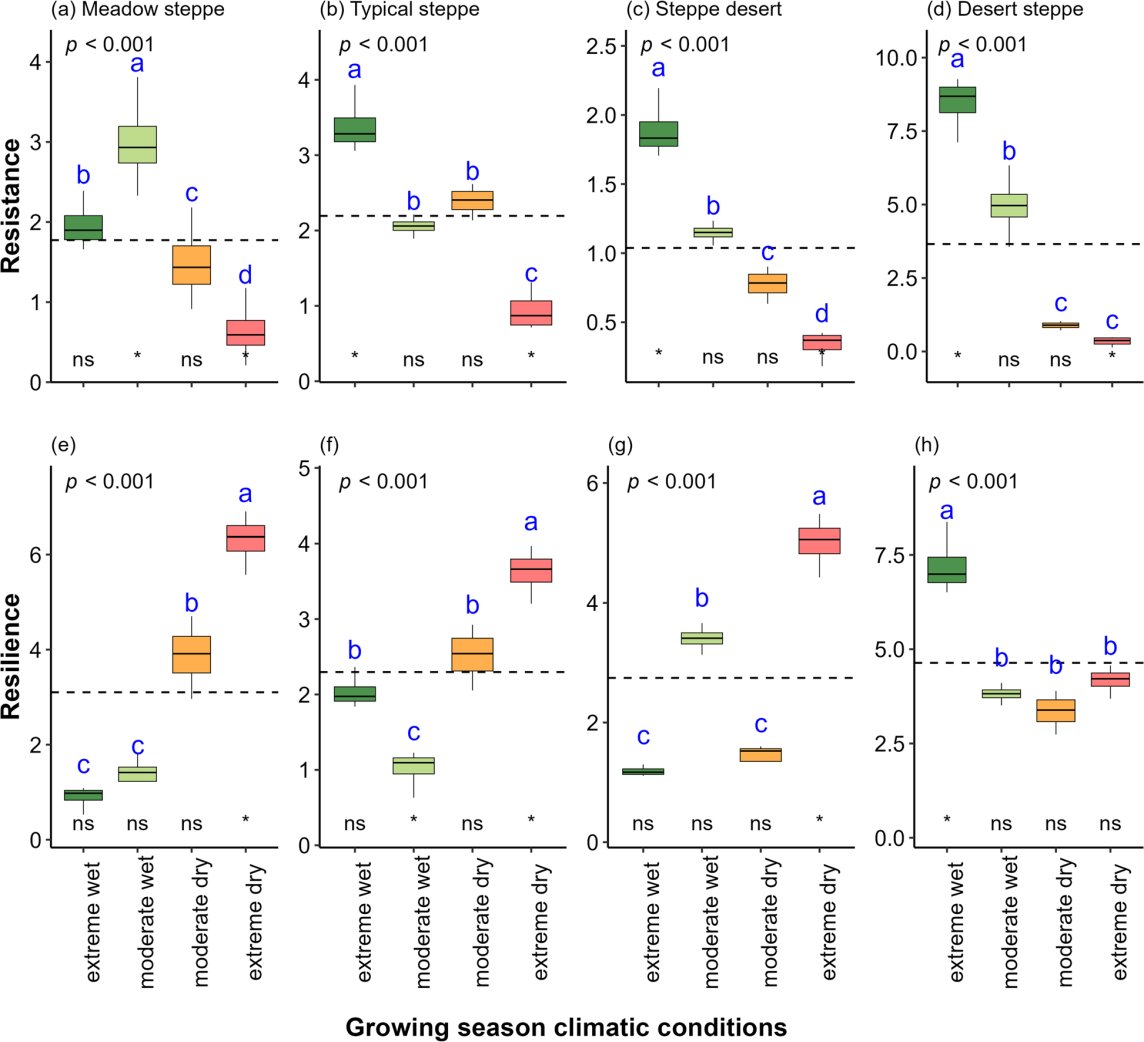
Ecosystem resistance (**a**-**d**) against and resilience (**e**–**h**) towards growing season climate extremes (SPEI-6) for meadow steppe (**a** & **e**), typical steppe (**b** & **f**), steppe desert (**c** & **g**), and desert steppe (**d** & **h**). The *p* values represent the significant difference in resistance and resilience of annual net primary productivity (NPP) between four intensities of growing season wet and dry climate. Letters (a-d) on the boxes show significant differences in resistance and resilience of the annual NPP between different climatic conditions. The 25th (75th) and 50th percentiles are represented by the lower (upper) quartiles and the horizontal line close to the centre of the boxes, respectively

**Fig. 17:**
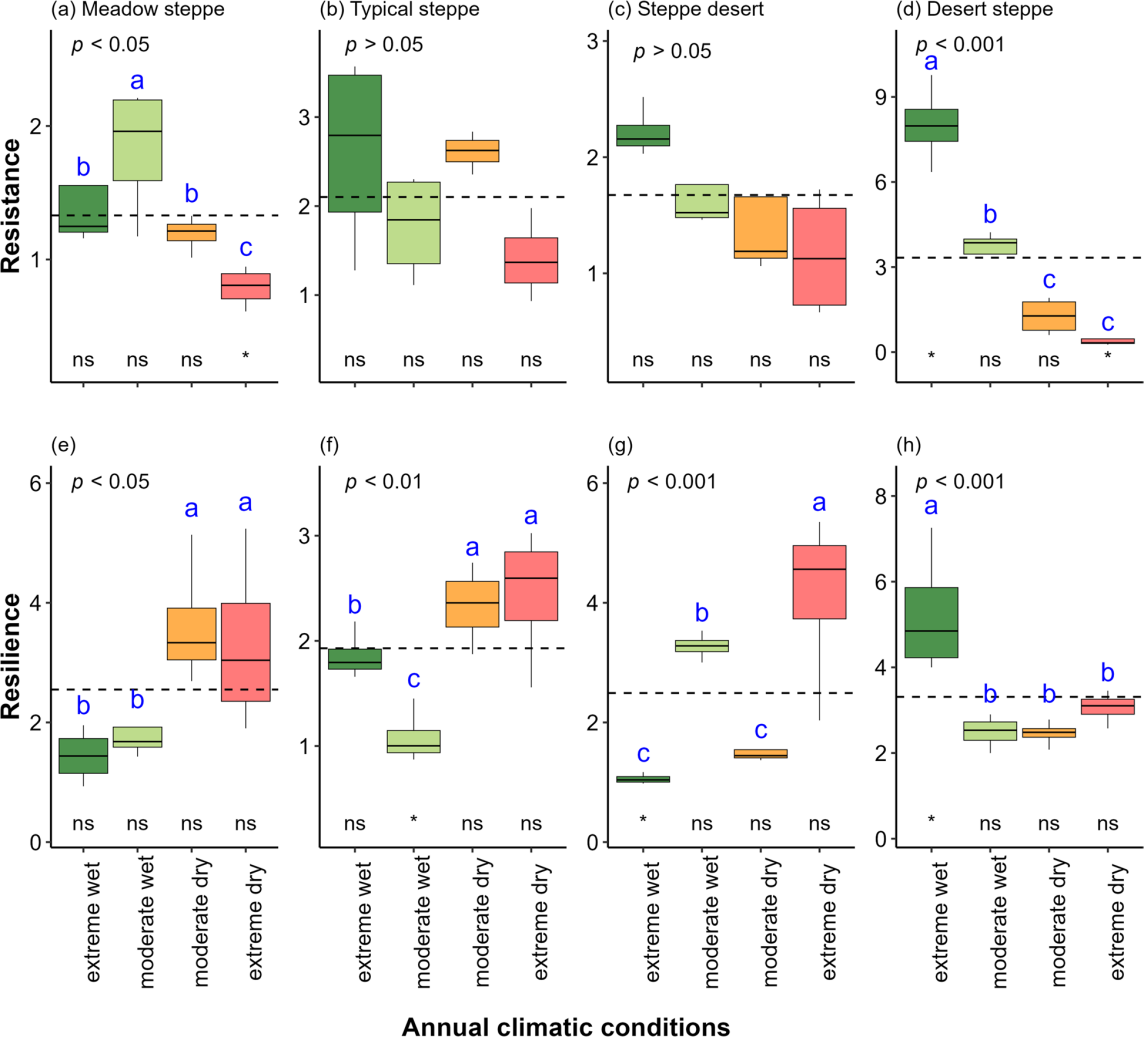
Ecosystem resistance (**a**-**d**) against and resilience (**e**–**h**) towards annual climate extremes (SPEI values in 1-Year) for meadow steppe (**a** & **e**), typical steppe (**b** & **f**), steppe desert (**c** & **g**), and desert steppe (**d** & **h**). The *p* values represent the significant difference in resistance and resilience of annual net primary productivity (NPP) between four intensities of annual wet and dry climate. Different letters (a-c) on the boxes show significant differences in resistance and resilience of the annual NPP between different climatic conditions. The 25th (75th) and 50th percentiles are represented by the lower (upper) quartiles and the horizontal line close to the centre of the boxes, respectively

Ecosystem resistance of the four grassland types was assessed against four extreme intensities of annual and growing season climates (Figs. 16a-d and 17a-d). Growing season and annual climate extreme intensities (moderate and extreme) and directions (wet and dry) were identified using SPEI values: moderate dry (extreme dry): > −1.28 to ≤ −0.67 (≤ −1.28), and moderate wet (extreme wet): ≥ 0.67 to < 1.28 (≥ 1.28) of 6-month and 12-month, respectively.

Resistance of grasslands showed large variations across four climate extreme intensities and the difference of resistance for all growing season climate extreme intensities was significant for all grasslands (Fig. 16a-d, *p* < 0.001). Irrespective of grassland types, grassland showed higher resistance against wet events, of which desert steppe, steppe desert and typical steppe exhibited higher resistance against extreme wet (Fig. 16b-d) and meadow steppe vegetation was highly resistance against moderate wet conditions (Fig. 16a). However, all four grassland types showed lower resistance against extreme dry conditions (Fig. 16a-d). Compared to the base-mean, grassland resistance was significantly lower against extreme dry and significantly higher against extreme wet conditions for all grasslands (all *p* < 0.05), except for resistance against wet events in meadow steppe (Fig. 16a-d). Like growing season climate extremes, resistance of grasslands in meadow and desert steppes exhibited significant differences between annual climate extreme intensities (both *p* < 0.05), where resistance was higher against wet events and lower against dry events (Fig. 17a and d). Although annual dry events lowered and wet events enhanced vegetation resistance in typical steppe and steppe desert, no significant differences were observed between the four climate extreme intensities in these two grassland types (Fig. 17b and c, both *p* > 0.05).

### Ecosystem resilience towards climate extremes

Ecosystem resilience showed considerable variations across the four climate extreme intensities, and the difference in resilience for all dry and wet episodes of growing season climate was significant for the studied grasslands (Fig. 16e-h, all *p* < 0.001). Examination of the resilience of grasslands towards growing season climate extremes revealed that resilience was notably high after dry conditions, particularly after extreme dry climate in the steppe desert, typical steppe, and meadow steppe (Fig. 16e-g). However, these grasslands demonstrated lower resilience after wet conditions compared to dry events. Unlike these grasslands, desert steppe showed the highest resilience towards extreme wet climate compared to other climate events (Fig. 16h). Compared to the base-mean, the resilience of steppe desert, typical steppe and meadow steppe was significantly higher after extreme dry climate. Like ecosystem resilience towards growing season climate extremes, all these grasslands showed a similar pattern of resilience towards annual climate extremes (Fig. 17e-h, all *p* < 0.05). The main differences in vegetation resilience between annual and growing season climate extremes are that resilience towards growing season dry climate increased with increasing drought intensity (Fig. 16e and f), while resilience towards annual dry climate intensities remained the same for meadow steppe and typical steppe (Fig. 17e and f). No detectable discrepancy was observed for the pattern of vegetation resilience of steppe desert, and desert steppe towards growing season and annual climate extreme intensities. That is, resilience was lowest (highest) towards extreme wet (extreme dry) conditions in steppe desert and highest (lowest) towards extreme wet (dry) conditions in desert steppe for growing season and annual climate extremes (Figs. 16g and h, 17g and h).

## Discussion

The NPP of terrestrial ecosystems is crucial for assessing their functionality and sustainability, impacting biogeochemical cycles and climate change mitigation [51]. However, recent climate anomalies have posed substantial challenges to the functioning and stability of grasslands, leading to declines in NPP [2, 4, 92]. Understanding the impacts of extreme climate events on grassland NPP has become a critical research priority, especially given the rising occurrence and severity of such events. Research indicates that NPP responses to climatic perturbations vary among grassland types [38, 93, 94]. This paper focuses on (i) the spatiotemporal patterns of annual NPP in grasslands and the underlying determinants of NPP dynamics, (ii) ecosystem resistance against climate extremes, and (iii) ecosystem resilience towards climate extremes across four climatic episodes and four grassland types in Inner Mongolia for the period 2000–2019. This study provides evidence of increasing grassland productivity in Inner Mongolian grasslands, despite the differing resistance and resilience among these grassland types.

### Spatiotemporal changes in NPP and underlying reasons

This study revealed a 20-year upward trend in annual NPP across four grassland types in Inner Mongolia, demonstrating both spatial and temporal increase in NPP throughout the region (Figs. 8 and 9). This finding aligns with several studies confirming increased productivity in Chinese grasslands over recent decades. For example, an upward trend in NPP was observed for (i) steppe grasslands (i.e. meadow, typical and desert steppes) in Inner Mongolia over 1980–2006 [94], (ii) several grasslands in Gansu province, China over 1982–2011 [51], (iii) grasslands in Gannan prefecture from 2000 to 2010 [45, 46], and (iv) steppe desert, and other steppe grasslands (typical and meadow steppes) in Inner Mongolia over 2000–2019 [38]. Using experimental observations, Zhao et al [95] also reported increasing trends in primary productivity in steppe desert, and desert and meadow steppes in Inner Mongolia for the period 2005–2012. When comparing the upward trend of NPP in our study with other vegetation indices used in previous studies, our findings are coherent with earlier findings. For example, an increasing NDVI trend was observed in temperate grasslands in China between 1982 and 1999 [96], in Inner Mongolian grasslands between 1982 and 2013 [97], and in cold steppe ecoregions in China between 1982 and 2012 [42].

The observed significant increasing trend in annual NPP across grassland types was driven by a combination of rising GSP and AP, alongside improving water balance (reflected in higher AI and SPEI values). These trends indicate a regional shift toward wetter conditions, alleviating historical moisture constraints on productivity (Fig. 10). The persistence of significant NPP-precipitation relationships after controlling for CO_2_ provides strong evidence that water availability is a genuine driver of grassland productivity, rather than a spurious correlation driven by shared temporal trends with rising CO_2_. The minimal reduction in correlation strength after accounting for CO_2_ further supports the robustness of precipitation as the primary limiting factor in these water-limited ecosystems [49].

Notably, the increasing AI reflects greater water availability relative to demand, rather than heightened dryness. The documented positive correlations between NPP and precipitation, AI and SPEI in our study are consistent with previous studies in arid and semi-arid grasslands [47, 49, 98–100]. For example, enhanced precipitation has been shown to increase (i) NPP in alpine grasslands on the Tibetan Plateau (Sun et al. 2019), (ii) NDVI in grass-dominated steppes in Yunnan Province, China [101], and (iii) aboveground biomass production across diverse grassland ecoregions, including dry steppe, humid temperate, and savanna [49].

The concurrent use of SPEI and AI in this study, rather than being redundant, illuminates different aspects of grassland responses to water availability. The consistent positive NPP-SPEI correlations across all grassland types (Fig. 13) demonstrate a universal principle: all grasslands increase productivity during wetter-than-normal periods [2, 4], regardless of their baseline aridity. The significant, positive NPP-AI correlations were only observed in arid grasslands (steppe desert and desert steppe; Fig. 14), suggesting that the productivity of these chronically water-limited ecosystems increased linearly with moisture availability. In contrast, no significant correlations between NPP and AI in semi-arid grasslands suggest they operated closer to optimal moisture conditions, where additional water yielded diminishing productivity returns—a classic saturating response [98].

However, we did not find any significant correlations between NPP and temperatures (GST and MAT), except for a significant negative correlation between NPP and GST_max_ in the meadow steppe. The static to insignificant negative correlations between NPP and temperature variables across the four grasslands are likely due to deficient soil moisture and high PET. This was further confirmed by analyzing the relationships of NPP with SPEI and AI. The lowest NPP values were observed at the lowest SPEI and AI values, suggesting that a decrease in water availability, coupled with an increase in PET, leads to higher plant mortality and reduced seed germination [102]. Although increasing vegetation productivity with rising precipitation variability was recorded in our study and in recent studies [5, 103], excessive GST and MAT and recurring dry episodes might hinder the functioning of plant communities in Inner Mongolia [38]. This impact has been examined through SPEI and NPP analyses (Figs. 16 and 17), demonstrating the resistance and resilience of particular grassland type under various climate extremes.

Beyond the climatic (precipitation) and atmospheric (CO_2_ fertilization) drivers, anthropogenic management factors may have contributed to observed NPP increases across Inner Mongolian grasslands. Specifically, livestock numbers in Mongolia declined by approximately 25–30% over the past two decades due to economic transitions and government policies [104]. This reduction in grazing pressure likely facilitated biomass accumulation by reducing vegetation removal [105]. In addition to reduced grazing intensity, large-scale ecological restoration programs have played a complementary role. For instance, the "*Returning Grazing Lands to Grasslands*" program, initiated in 2003, has substantially increased grassland coverage, leading to a mean rate of aboveground biomass carbon stock increase of 11 g m^−2^ yr^−1^ and belowground biomass carbon stock increase of 32 g m^−2^ yr^−1^ [105]. These findings are consistent with experimental studies reporting that grazing exclusion can increase aboveground biomass by 30–50% within a decade in Inner Mongolian grasslands [106], with similar effects observed in other grassland ecosystems such as the Qinghai-Tibet Plateau [107].

Importantly, management effects might interact with climate: reduced grazing amplifies wet-year productivity gains while buffering drought impacts [107]. Although we cannot quantitatively partition these effects without spatially explicit grazing and policy data, the persistence of significant NPP-precipitation relationships after controlling for CO_2_ and the clear aridity gradient patterns supports water availability as the primary driver ([98, 107, 108]), with management playing a secondary modulating role [109].

### Vegetation resistance and resilience

Understanding ecosystem resistance against and resilience towards climatic perturbations is critical to forecasting the effects of future climatic episodes (e.g., heatwaves and droughts) on ecosystem functioning [80] and stability [2]. The observed low resistance of all grassland types against extreme dry climatic conditions in our study likely stems from shortage of water availability during the growing season. The steppe desert, and desert steppe, classified as arid ecosystems (AI: 0.03–0.2), along with the semi-arid meadow steppe and typical steppe (AI: 0.2–0.5), already operate near their hydrological thresholds. Under such baseline water stress conditions, intensified dry episodes trigger to stomatal closure to prevent water loss, reduction of photosynthesis and eventual NPP collapse due to carbon starvation.

Previous empirical studies reported that species richness in semi-arid steppe (meadow steppe and typical steppe) range between 2 and 28 species m^2^ [110], and in arid steppe (steppe desert and desert steppe) ranges between 2 and 11 species m^2^ [110]. In our field observation in 2018, we recorded species richness varies from 9–14 species m^2^ in meadow steppe (wet condition) to 7–17 species m^2^ in typical steppe (normal climatic condition) to 5–10 species m^2^ in steppe desert (normal climatic condition). The observed significant positive species richness-NPP relationships in these three grasslands suggests that grassland productivity under favorable climatic conditions increase with gradient of species richness. We have recorded various dominant and sub-ordinate species in these grasslands.

The observed higher resistance against wet climatic conditions in all our grasslands could be resulted from the presence of dominant and subordinate species belonging to different functional groups (i.e., grass, herbs, legume and sedge). Dominant species use moisture under water surplus conditions while suffering from water scarce condition, ultimately affecting the total NPP. The findings of our study are well-supported by existing literature that highlights the distinct characteristics of plant communities in desert steppe and steppe desert ecosystems. These ecosystems are primarily composed of perennial grasses, xerophyte herbs, and shrubs, such as *A. polyrrhizum*, *S. glareosa*, *S. gobica*, *C. sinica*, and *A. xerophytica*. These species exhibit high resistance to wet conditions due to their deep root systems and water-retaining capacities, which enhance their overall adaptability ([58], Song et al. 2021). Moreover, meadow steppe and typical steppe ecosystems are dominated by herbaceous plants like *F. lenensis*, *S. baicalensis*, *P. attenuata*, and *L. chinensis*, which are primarily low-anchored species. These species exhibit lower resistance to droughts, making them more vulnerable to the impacts of dry climatic events [58–60]. Under resource-scarce conditions, these grasslands demonstrated reduced resistance to extreme dry climates, as drought-induced stress creates resource competition that adversely affects subordinate and stress-sensitive species, leading to competitive exclusion [36].

Another possible mechanism for the reduced resistance of our studied grasslands against extreme dry climate could be attributed to (i) recurring dry climatic conditions reduce soil moisture recharge, causing adverse abiotic conditions for plants in the growing season (Wu et al. 2002), and (ii) and existence of stress-intolerant plant functional groups and species [111]. In Inner Mongolian grasslands, previous studies have identified forbs and grasses as the dominant functional groups [58, 63]. This observation aligns with our field investigation, which confirms that forbs and grasses are dominant in the meadow steppe, typical steppe, and steppe desert. For instance, *Stipa grandis, S. krylovii*, and *Leymus chinensis* are dominant species within the functional group grasses, while *Artemisia frigida* is the dominant forb in the meadow steppe. Similarly, in the typical steppe, dominant grasses include *Poa attenuata, Festuca lenensis, Stipa baicalensis*, and *Leymus chinensis*, while *Filifolium sibiricum*, and *Artemisia frigida* are the dominant forbs. The lower resistance of these grasslands against dry climatic conditions could be resulted from the prevalence of forbs, which are less drought-resistant compared to other functional groups like sedge and legume 112. Forbs rely on deeper bases of moisture to compensate for their reduced water use efficiency during mild dry climatic conditions [113]. With the growing strength of dry conditions (i.e., from moderate dry to extreme dry) and the occurrences of multi-year droughts [1], soil moisture from the deeper soil layers might dry out, resulting in a decline in the richness and mortality of plants belonging to forbs. Consequently, this could explain the observed lower resistance of our studied grasslands against extreme dry compared to moderate dry climatic conditions.

Multiyear droughts progressively deplete soil moisture reserves and plant carbohydrate stores, leading to hydraulic failure, root mortality and seed bank exhaustion, which collectively erode grassland resistance beyond the capacity for single-year dry event resistance. In our study, we recorded 3 multi-year dry climatic conditions in meadow steppe, 4 in typical steppe, 3 each in steppe desert and desert steppe. Consistent with our SPEI time series (Figs. 6 & S3), multiyear droughts have been reported to increase in Mongolia [10] and globally [1], which may lead to lower resistance in grasslands. For example, using NDVI and SPEI, Chen et al [1] reported that multiyear droughts in Mongolia caused considerable declines in vegetation greenness. Consistent with these observations, our results reveal the lowest mean annual NPP values during the multiyear dry climatic conditions across all four grassland types. For example, the mean annual NPP in meadow steppe dropped from 241 g C m^−2^ yr^−1^ (20 years average) to 226 g C m^−2^ yr^−1^ during multiyear dry periods. Similarly, for typical steppe, mean NPP (long-term average) declined from 160 g C m^−2^ yr^−1^ to 149 g C m^−2^ yr^−1^ under multiyear dry climatic conditions. These reductions highlight the compounding effects of consecutive dry years, which exceed the resistance capacity of these grasslands compared to isolated dry climatic conditions [1].

Examination of the resilience of the four grassland types in our study showed an opposite pattern, that is, lower resilience towards wet conditions and higher resilience towards dry climatic conditions (Figs. 16e-h and 17e-h). This apparent pattern most likely resulted from several mechanisms (e.g. plant-environment, and plant-soil-environment interactions). For instance, after dry conditions, the complementarity between dominant and subordinate species, as well as between shallow- and deep-rooted species, may enhance overall ecosystem resilience. Herbaceous plants like *F. lenensis*, *S. baicalensis*, *P. attenuata*, and *L. chinensis* are low-anchored species, which are dominated in typical steppe and meadow steppe in Inner Mongolia. Although these low-anchored species affected by extreme dry conditions [58–60], they recover quickly when normal climatic conditions return.

The higher resilience and resistance of vegetation in desert steppe to extreme wet climate suggest that such events are favorable for the vegetation in these grasslands. Grasslands in desert steppe typically have a low groundwater availability because of low precipitation (101 mm yr^−1^); thus, precipitation is the key determinant of productivity [97]. This is evident in our study when the resistance of desert steppe is considered. Grasslands in desert steppe have developed adaptation mechanisms to cope with climate-induced stresses [114]. However, prolonged dryness makes it more difficult for the ecosystem to absorb the shocks, thus showing lower resistance against drought. Our study confirmed that excessive dry climatic conditions had a significantly higher impact on grassland ecosystems than the lower intensity droughts (e.g. moderate dry climate), which is coherent with previous studies in hay meadow in Europe [4], Inner Mongolian grasslands [38], and grasslands in Southwest China (Li et al. 2019a).

The findings of the resistance and resilience of steppe desert, typical steppe and meadow steppe in our study confirm that vegetation resilience and resistance of these grasslands are inversely correlated. This study supports the notion that higher (lower) resistant ecosystem exhibits lower (higher) resilience, which is in line with some recent studies across different grassland types [4, 115]. Given the anticipated future intensifications in the strength and occurrence of weather events [116], this study may assist in identifying grasslands that are most vulnerable to lowering ecosystem functioning and stability. These sensitive grasslands would require appropriate management and conservation initiatives under the accelerating rate of intense climatic perturbations. The scientific evidence of resistance and resilience during different dry and wet climatic episodes across the four investigated grassland types in Inner Mongolia equips stakeholders and land managers with valuable insights. This information can be leveraged to inform adaptive strategies and strategic planning aimed at ensuring stable resource delivery and restoring degraded grassland ecosystems, particularly steppe grasslands in the region.

Despite a robust understanding of grassland ecosystem stability under frequent and intense climate extremes, several shortcomings exist in this paper. There are numerous variables that contribute to ecosystem functioning, including soil physico-chemical properties and management practices (e.g., grazing exclusion). An integrated study in the future combining all these factors could explain the dynamics and stability in Inner Mongolian grasslands. Although this study utilized MODIS NPP data with a spatial resolution of 500 m, alternative vegetation indices, such as leaf area index with better spatial resolution (e.g., 10 m), could enhance the exploration of resilience and resistance in grassland ecosystems, as NPP changes may not fully capture the complexities of biodiversity characteristics and their influence on resilience. As several extreme events can occur simultaneously (e.g., occurrence of drought and heat wave in a particular growing season), the understanding of how ecosystem functioning responds to combined climatic events will be of great significance for sustainable management of grassland ecosystems. While our power analysis and sensitivity analysis confirmed statistical robustness, we acknowledge that longer time series would provide greater confidence in stability metrics and enable detection of rarer events (e.g., extreme wet conditions following multi-year droughts). Despite these few shortcomings in this study, the findings of divergence of grassland resistance and resilience provide a comprehensive understanding of the stability of grasslands in Inner Mongolia.

## Conclusions

Despite interannual fluctuation in NPP, all grassland types exhibited significant increasing NPP trends (2000–2019), with meadow steppe showing the highest productivity (251.65 g C m^−2^ yr^−1^) and growth rate (4.54 g C m^−2^ yr^−1^), compared to other three grassland types. This study revealed a clear precipitation-driven productivity dynamic, where increased GSP and AP significantly enhanced NPP across all grassland types and the partial correlation analysis confirmed that these positive NPP-precipitation relationships remained significant after controlling for rising CO_2_ levels. Productivity was strongly tied to climatic conditions, with NPP showing positive correlations with SPEI and negative associations with temperature extremes (e.g., GST_max_). Notably, arid grasslands (steppe desert and desert steppe) benefited from increased moisture availability, as evidenced by positive NPP-AI relationships, while semi-arid grasslands (meadow and typical steppe) displayed no significant responses, suggesting optimal moisture thresholds. Species richness further enhanced NPP in the investigated grassland types in 2018, underscoring the stabilizing role of biodiversity.

Examination of grassland stability revealed that wet (dry) climatic conditions exhibited higher (lower) resistance for typical steppe, meadow steppe and steppe desert. The lower resistance but higher resilience of NPP after extreme dry climatic conditions in steppe desert, and typical and meadow steppes suggest that vegetation in these three grasslands is very sensitive to extreme drought, while they recover faster after extreme dry climate compared to the recovery after wet conditions. This paper concludes that the variations of ecosystem resistance and resilience are inversely correlated and are not only subjected to climate extreme intensities and directions but also depend on vegetation health in different grassland types and compounding effects of multiyear dry periods. The scientific evidence of the resistance and resilience of ecosystems under different dry and wet climatic episodes across the investigated four grassland types in Inner Mongolia provides stakeholders and land managers with evidence that can be utilized to develop adaptation and strategic planning for achieving stable resource delivery and restoring degraded grassland ecosystems, particularly steppe grasslands in Inner Mongolia.

## Supplementary Information

Supplementary Material 1.

## Data Availability

The datasets generated and/or analysed during the current study are available in the https://doi.org/10.5067/MODIS/MOD17A3HGF.006 (annual NPP); https://doi.org/10.5067/MODIS/MOD11A1.061 (temperature); https://doi.org/10.1038/sdata.2015.66 (precipitation) and https://doi.org/10.15138/wkgj-f215 (CO2 concentration).

## Abbreviations

CHIRPS: Climate Hazards Center InfraRed Precipitation with Stations
MODIS: Moderate Resolution Imaging Spectroradiometer
NPP: Net Primary Productivity
SPEI: Standardized Precipitation Evapotranspiration Index
GSP: Growing Season Precipitation
AP: Annual Precipitation
AI: Aridity Index
GST_max_: Growing season maximum temperature
GST_min_: Growing season minimum temperature
GST_mean_: Growing season temperature
MAT_mean_: Mean Annual Temperature
MAT_max_: Mean Annual Maximum Temperature
MAT_min_: Mean Annual Minimum Temperature
