# Supplementary material for: Enhanced net primary productivity across Inner Mongolian grasslands despite diverging stability under climate perturbations
Source: BMC Plant Biol. 2026 Jun 5;26:1351. doi: 10.1186/s12870-026-09165-7 (PMC13459204; doi:10.1186/s12870-026-09165-7)
Supplement: Supplementary file 1 [file 12870_2026_9165_MOESM1_ESM.pdf]

Supplementary Information for

Enhanced net primary productivity across Inner Mongolian grasslands despite diverging stability under climate perturbations

Md Lokman Hossain [1,*] and Derrick Y. F. Lai [1,*]

[1]Department of Geography and Resource Management, The Chinese University of Hong Kong, Shatin, New Territories, Hong Kong, China

[*]Correspondence: Md Lokman Hossain (lokmanbbd@gmail.com); Derrick Y. F. Lai (dyflai@cuhk.edu.hk)

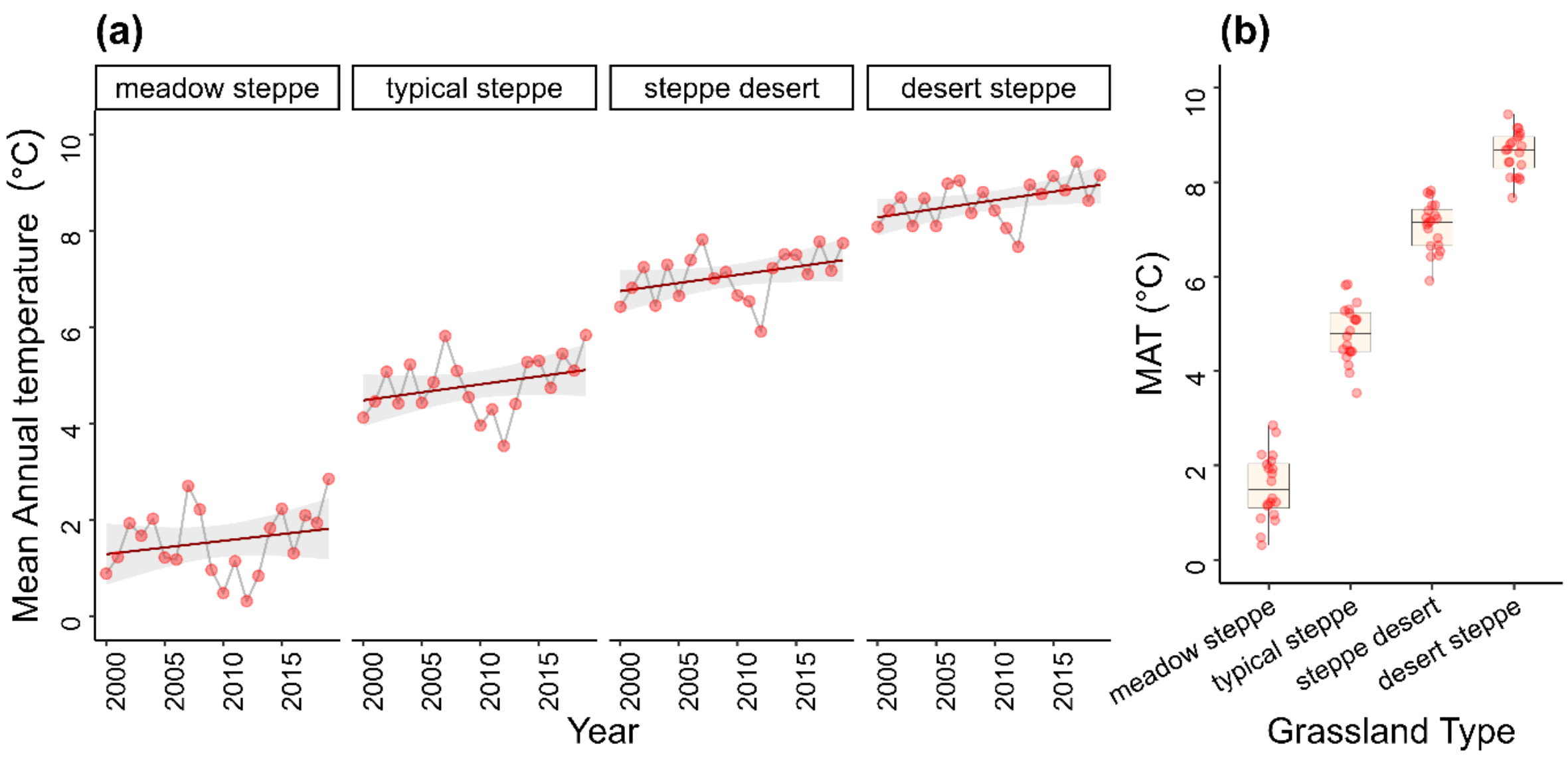

Fig. S1 Temporal trends (**a**) and variations (**b**) of Mean Annual Temperature (MAT) in meadow steppe, typical steppe, steppe desert and desert steppe for the period 2000-2019. Positive tau (τ) values indicate the strength and direction of the monotonic increase in MAT over the specified period. The *p* values denote the level of significance of the increasing trends. The solid blue lines represent the trend line of MAT. The confidence intervals (95%) for fluctuations in MAT are indicated as bands along the lines.

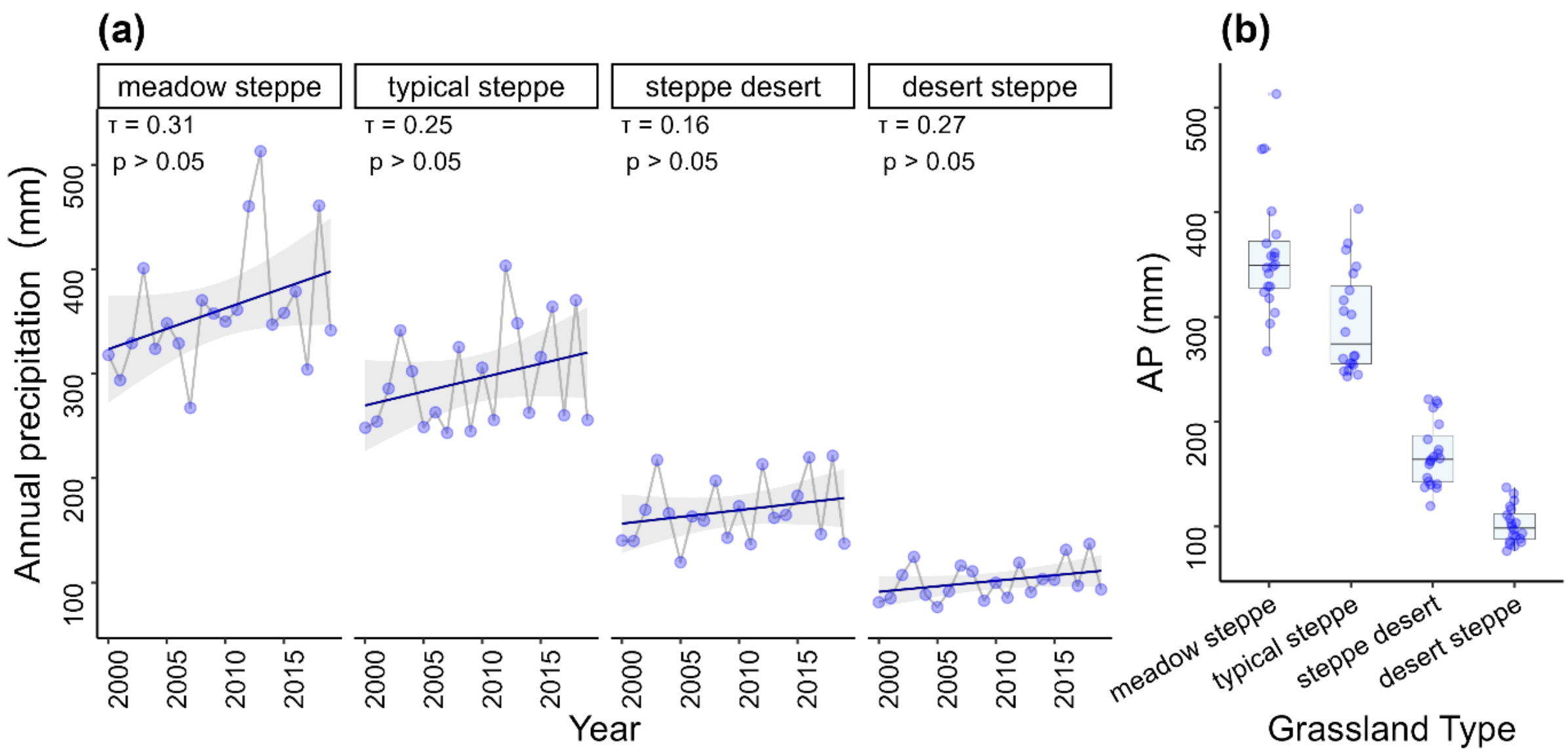

Fig. S2 Temporal trends (**a**) and variations (**b**) of annual precipitation (AP) in meadow steppe, typical steppe, steppe desert and desert steppe for the period 2000-2019. Positive tau (τ) values indicate the strength and direction of the monotonic increase in AP over the specified period. The *p* values denote the level of significance of the increasing trends. The solid blue lines represent the trend line of AP. The confidence intervals (95%) for fluctuations in AP are indicated as bands along the lines.

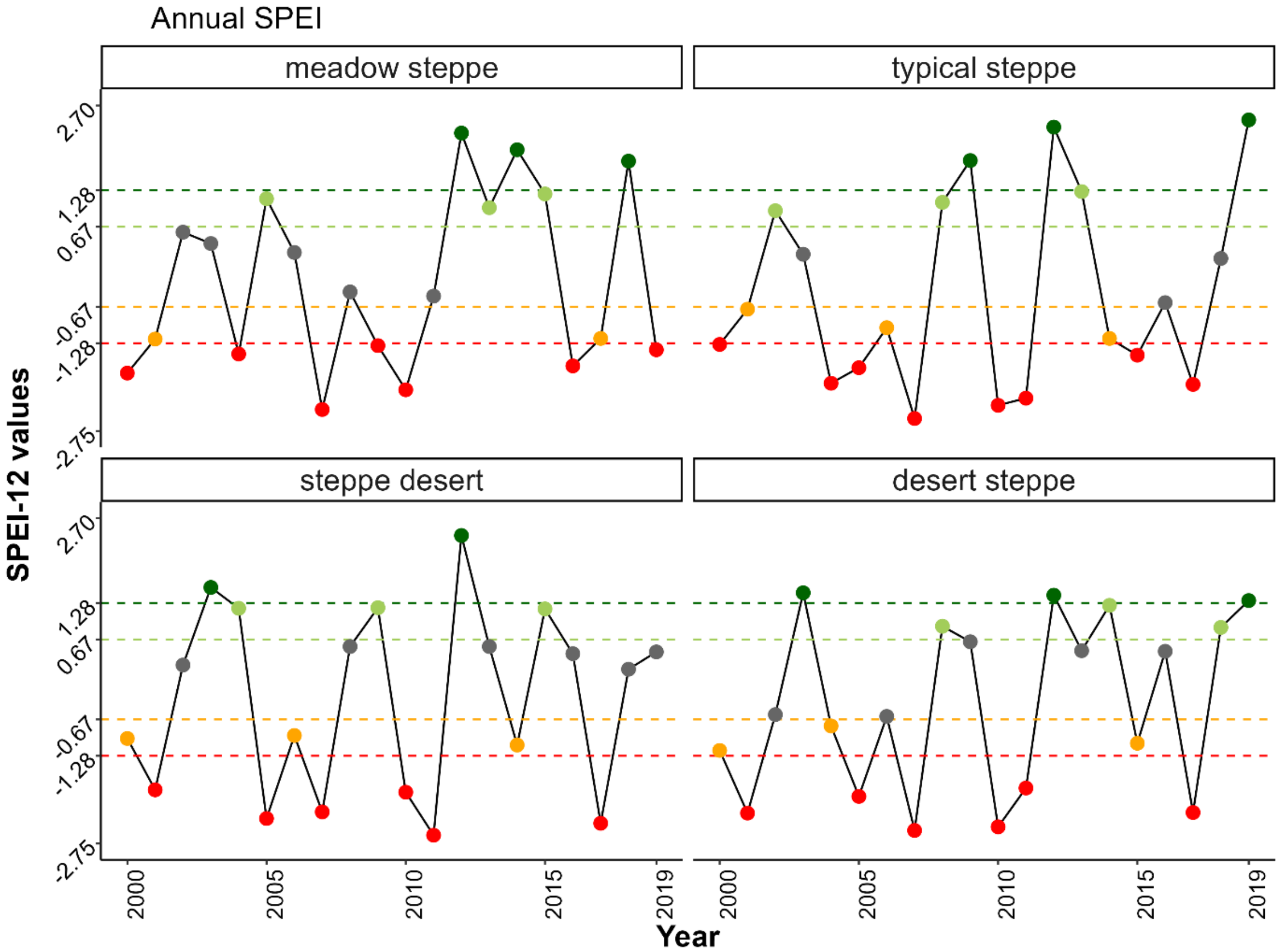

Figure S3. The number of occurrences of annual climatic events (extreme wet, moderate wet, normal, moderate dry, and extreme dry) based on 12-months (October-September) standardized precipitation evapotranspiration index (SPEI) values for four grassland types (meadow steppe, typical steppe, steppe desert and desert steppe) in Inner Mongolia for the period 2000-2019. The dark olive green and dark green points indicate moderate wet and extreme wet conditions, while orange and red points denote moderate dry and extreme dry conditions. Normal climatic conditions are shown in grey points. The horizontal dashed lines indicate the cut-off values of annual SPEI in respective climatic conditions (dark green for extreme wet, dark olive green for moderate wet, orange for moderate dry, and red for extreme dry).

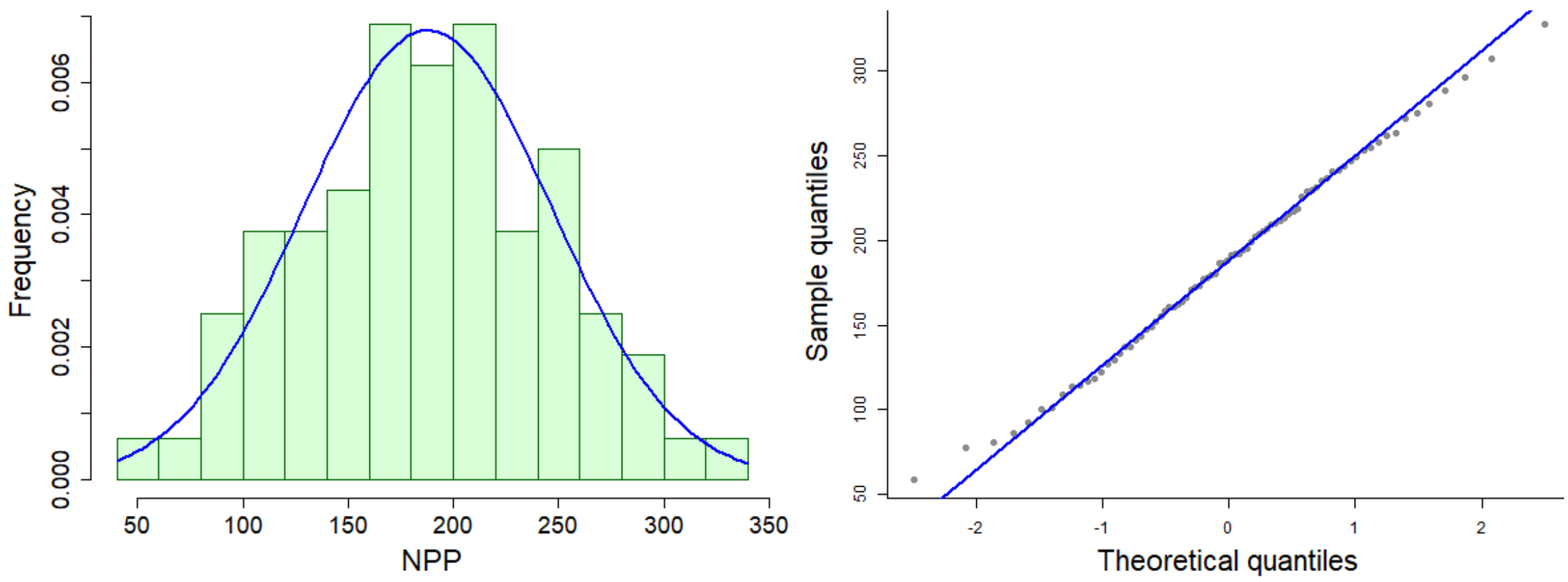

Figure S4. Normality assessment of the data using histogram and quantile-quantile plot.

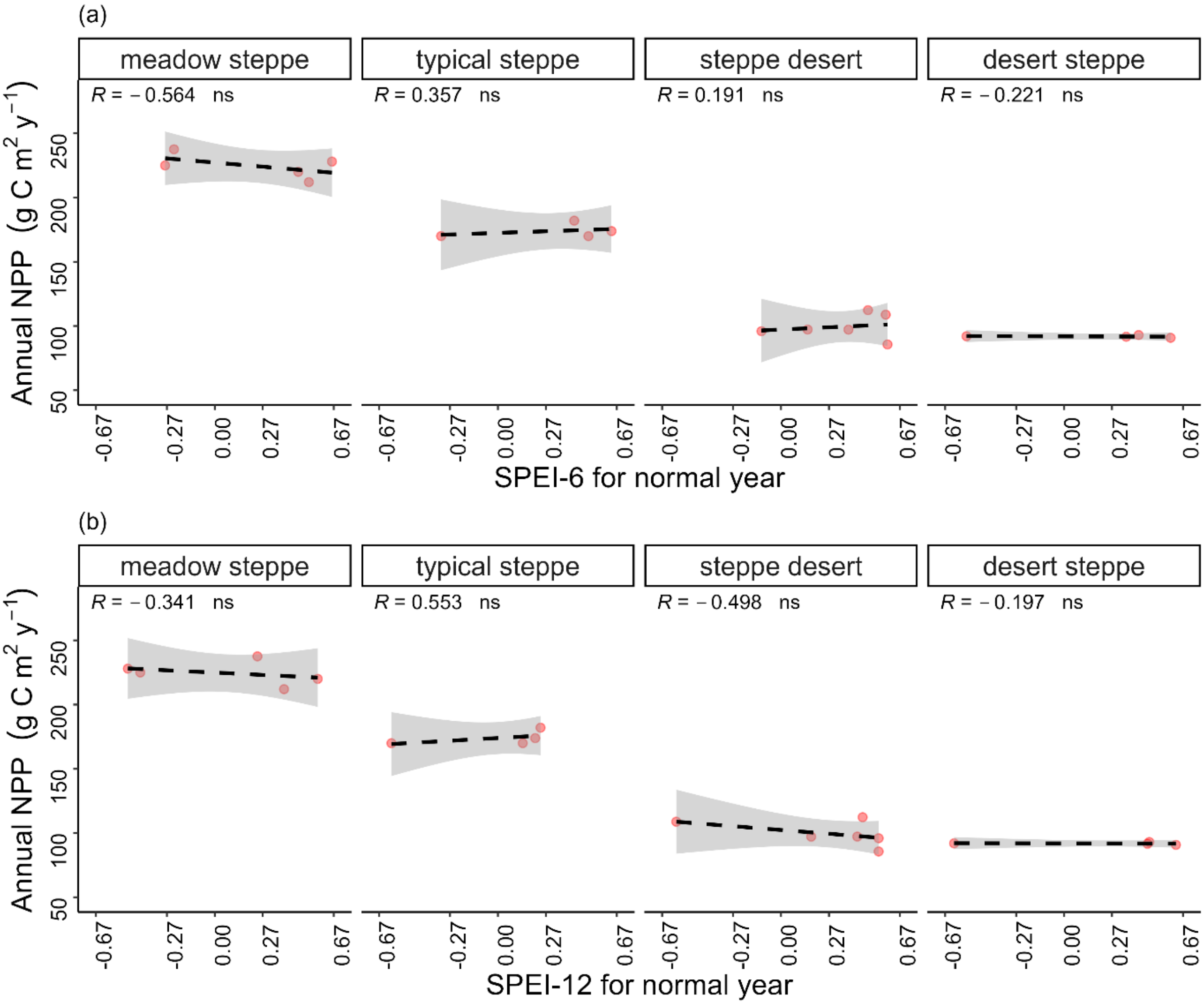

Figure S5. Correlation between annual net primary productivity (NPP) and growing season (**a**) and annual (**b**) climatic condition for the normal season/year for the four grassland types (meadow steppe, typical steppe, steppe desert, and desert steppe) in Inner Mongolia. Standardized precipitation evapotranspiration index (SPEI) values between >-0.67 and <0.67 were considered for normal climatic conditions. The correlation coefficient (*R* values) obtained by Pearson correlation analysis denotes the strength and direction of the linear relationship between annual NPP and SPEI values. The 'ns' indicate the insignificant relationship between annual NPP and SPEI values. The confidence intervals (95%) for relationships are indicated as bands along the dashed lines.

Table S1. Statistics of the autocorrelation check using the Durbin-Watson (DW) test. Residuals are considered uncorrelated when the DW statistic falls within the range of 1.5 to 2.5.

Statistics	Grassland types			
	Meadow steppe	Typical steppe	Steppe desert	Desert steppe
DW value	1.84	1.95	1.89	1.93
p value	0.07	0.24	0.09	0.33

Table S2. Post-hoc power analysis results for resistance comparisons across four grassland types in Inner Mongolia. The table shows comparisons between different climate extreme categories, including mean resistance values, standard deviations (SD), Cohen's d effect sizes, and statistical power for each comparison. Power ≥ 0.80 was considered adequate. CE1 = Climate extreme category 1; CE2 = Climate extreme category 2.

Grassland type	Comparison (CE1 vs CE2)	Mean (CE1)	Mean (CE2)	SD (CE1)	SD (CE2)	Cohen's d	Power	Adequate (≥ 0.80)
Meadow steppe	Extreme dry vs Extreme wet	0.642	1.961	0.4	0.314	3.668	0.989	Yes
	Extreme dry vs Moderate dry	0.642	1.492	0.4	0.53	1.809	0.573	No
	Extreme wet vs Moderate wet	1.961	3.001	0.314	0.611	2.139	0.714	No
Typical steppe	Extreme dry vs Extreme wet	0.942	3.389	0.274	0.38	7.382	1	Yes
	Extreme dry vs Moderate dry	0.942	2.391	0.274	0.207	5.964	1	Yes
	Extreme wet vs Moderate wet	3.389	2.056	0.38	0.132	4.68	1	Yes
Steppe desert	Extreme dry vs Extreme wet	0.336	1.891	0.108	0.213	9.221	1	Yes
	Extreme dry vs Moderate dry	0.336	0.776	0.108	0.116	3.926	0.995	Yes
	Extreme wet vs Moderate wet	1.891	1.148	0.213	0.074	4.673	1	Yes
Desert steppe	Extreme dry vs Extreme wet	0.341	8.44	0.161	0.939	12.017	1	Yes
	Extreme dry vs Moderate dry	0.341	0.887	0.161	0.132	3.701	0.99	Yes
	Extreme wet vs Moderate wet	8.44	4.958	0.939	1.133	3.346	0.974	Yes

Table S3. Post-hoc power analysis results for resilience comparisons across four grassland types in Inner Mongolia. The table shows comparisons between different climate extreme categories, including mean resistance values, standard deviations (SD), Cohen's d effect sizes, and statistical power for each comparison. Power ≥ 0.80 was considered adequate. CE1 = Climate extreme category 1; CE2 = Climate extreme category 2.

Grassland type	Comparison (CE1 vs CE2)	Mean (CE1)	Mean (CE2)	SD (CE1)	SD (CE2)	Cohen's d	Power	Adequate (≥ 0.80)
Meadow steppe	Extreme dry vs Extreme wet	6.305	0.892	0.558	0.25	12.519	1	Yes
	Extreme dry vs Moderate dry	6.305	3.873	0.558	0.734	3.73	0.991	Yes
	Extreme wet vs Moderate wet	0.892	1.34	0.25	0.462	1.206	0.301	No
Typical steppe	Extreme dry vs Extreme wet	3.623	2.038	0.321	0.229	5.691	1	Yes
	Extreme dry vs Moderate dry	3.623	2.515	0.321	0.375	3.176	0.96	Yes
	Extreme wet vs Moderate wet	2.038	1.012	0.229	0.264	4.158	0.998	Yes
Steppe desert	Extreme dry vs Extreme wet	5.007	1.188	0.443	0.084	11.979	1	Yes
	Extreme dry vs Moderate dry	5.007	1.388	0.443	0.328	9.29	1	Yes
	Extreme wet vs Moderate wet	1.188	3.404	0.084	0.218	13.396	1	Yes
Desert steppe	Extreme dry vs Extreme wet	4.172	7.215	0.369	0.811	4.832	1	Yes
	Extreme dry vs Moderate dry	4.172	3.351	0.369	0.5	1.87	0.6	No
	Extreme wet vs Moderate wet	7.215	3.814	0.811	0.245	5.681	1	Yes

Table S4. Summary of leave-one-out sensitivity analysis for resistance and resilience across all grassland types and climate extreme categories. The table shows the mean absolute percent change in stability metrics when removing each individual event year, and the percentage of analyses where qualitative conclusions remained unchanged after removal.

Metric	Mean absolute percent change	Overall percent conclusions unchanged
Resistance	5.22%	96.9%
Resilience	3.6%	93.8%
